# Rapid Generation of Circulating and Mucosal Decoy Human ACE2 using mRNA Nanotherapeutics for the Potential Treatment of SARS‐CoV‐2

**DOI:** 10.1002/advs.202202556

**Published:** 2022-10-10

**Authors:** Jeonghwan Kim, Antony Jozic, Anindit Mukherjee, Dylan Nelson, Kevin Chiem, Md Siddiqur Rahman Khan, Jordi B. Torrelles, Luis Martinez‐Sobrido, Gaurav Sahay

**Affiliations:** ^1^ Department of Pharmaceutical Sciences College of Pharmacy Robertson Life Sciences Building Oregon State University Portland OR 97201 USA; ^2^ High‐Throughput Screening Services Laboratory College of Pharmacy Oregon State University Corvallis OR 97331 USA; ^3^ Disease Prevention and Intervention and Population Health Programs Texas Biomedical Research Institute San Antonio TX 78227 USA; ^4^ Department of Biomedical Engineering Robertson Life Sciences Building Oregon Health & Science University Portland OR 97201 USA; ^5^ Department of Ophthalmology Casey Eye Institute Oregon Health & Science University Portland OR 97239 USA

**Keywords:** COVID‐19, gene therapy, human soluble ACE2, lipid nanoparticles, messenger RNA

## Abstract

Severe acute respiratory syndrome coronavirus 2 (SARS‐CoV‐2) can cause lethal pulmonary damage in humans. It contains spike proteins on its envelope that bind to human angiotensin‐converting enzyme 2 (hACE2) expressed on airway cells, enabling entry of the virus, and causing infection. The soluble form of hACE2 binds SARS‐CoV‐2 spike protein, prevents viral entry into target cells, and ameliorates lung injury; however, its short half‐life limits therapeutic utilities. Here, synthetic mRNA is engineered to encode a soluble form of hACE2 (hsACE2) to prevent viral infection. A novel lipid nanoparticle (LNP) is used for packaging and delivering mRNA to cells to produce hsACE2 proteins. Intravenously administered LNP delivers mRNA to hepatocytes, leading to the production of circulatory hsACE2 initiated within 2 h and sustained over several days. Inhaled LNP results in lung transfection and secretion of mucosal hsACE2 to lung epithelia, the primary site of entry and pathogenesis for SARS‐CoV‐2. Furthermore, mRNA‐generated hsACE2 binds to the receptor‐binding domain of the viral spike protein. Finally, hsACE2 effectively inhibits SARS‐CoV‐2 and its pseudoviruses from infecting host cells. The proof of principle study shows that mRNA‐based nanotherapeutics can be potentially deployed to neutralize SARS‐CoV‐2 and open new treatment opportunities for coronavirus disease 2019 (COVID‐19).

## Graphical abstract

A mRNA encoding human soluble angiotensin‐converting enzyme 2 (hsACE2) could be a therapeutic option to treat SARS‐CoV‐2 infection by inhibiting the virus entry. Lipid nanoparticles enable a rapid generation of circulating and mucosal hsACE2 protein by delivering the mRNA to the liver and lungs.

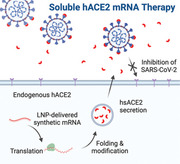

## 1 Introduction

More than 500 million cases with over 6 million deaths have resulted from coronavirus disease 2019 (COVID‐19).^[^ ^1^ ^]^ Severe acute respiratory syndrome coronavirus 2 (SARS‐CoV‐2), the pathogen responsible for COVID‐19, is a *β*‐coronavirus that primarily enters through the airways, affecting mainly the lungs. The envelope of SARS‐CoV‐2 is decorated with homotrimeric spike proteins that bind to the human angiotensin‐converting enzyme 2 (hACE2) receptor expressed on the host cell surface.^[^ ^2^, ^3^ ^]^ The spike protein is composed of S1 and S2 subunits responsible for viral attachment and fusion, respectively.^[^ ^4^ ^]^ Binding between the receptor‐binding domain (RBD), located within the S1 subunit, and hACE2 triggers a cascade that accelerates cellular entry and viral membrane fusion. hACE2 is expressed in the lungs, heart, kidney, and intestine for maintaining blood pressure in the Renin Angiotensin Aldosterone System (RAAS).^[^ ^5^ ^]^ It functions as a carboxypeptidase that converts Angiotensin 1 to Angiotensin (1‐9) or Angiotensin II to Angiotensin (1‐7), and both products are vasodilators with cardioprotective effects through the regulation of blood pressure.^[^ ^6^ ^]^ SARS‐CoV‐2 interacts with hACE2 to enter and infect human airway epithelial cells, causing cytotoxic responses. It can cause development of pneumonia and cytokine storm, leading to Acute Respiratory Distress Syndrome (ARDS) in severe cases.^[^ ^7^, ^8^, ^9^ ^]^ Once the virus infiltrates systemic circulation, it can dysregulate the RAAS and the immune system, cause endothelial cell damage, target other tissues that express hACE2, driving a multisystem organ failure.^[^ ^10^ ^]^ hACE2 consists of three segments: an extracellular segment that contains the peptidase domain to where the RBD binds, a transmembrane segment, and an intracellular segment.^[^ ^3^ ^]^ hACE2 can be cleaved by peptidases at the neck region of the extracellular segment, releasing a soluble form of hACE2 (hsACE2) which is enzymatically active.^[^ ^11^, ^12^ ^]^ Considering the high‐affinity binding of SARS‐CoV‐2 spike protein to hACE2 receptors,^[^ ^13^ ^]^ the soluble form (hsACE2) could prevent the virus from binding to the receptors through competitive inhibition.^[^ ^11^, ^12^ ^]^ A recent study indicates that recombinant hsACE2 protein could inhibit the SARS‐CoV‐2 infection in cultured kidney organoids.^[^ ^14^ ^]^ However, the relatively short half‐life of the recombinant hsACE2 in the bloodstream would require repeated administration to ensure the protein's long‐term circulation for days after exposure to SARS‐CoV‐2.^[^ ^15^ ^]^ Chimeric hsACE2, which contains a fused Fc region of human IgG, reduced viral infection in vitro; however, its pharmacokinetic benefit remains undetermined.^[^ ^16^, ^17^ ^]^ A short half‐life of hsACE2 (<2 h in mice) severely limits its time window of action where the extended residence of hsACE2 is desirable to mitigate SARS‐CoV‐2 mediated RAAS activation and hence to reduce inflammation‐related injury of organs.^[^ ^18^ ^]^

**Figure 1:**
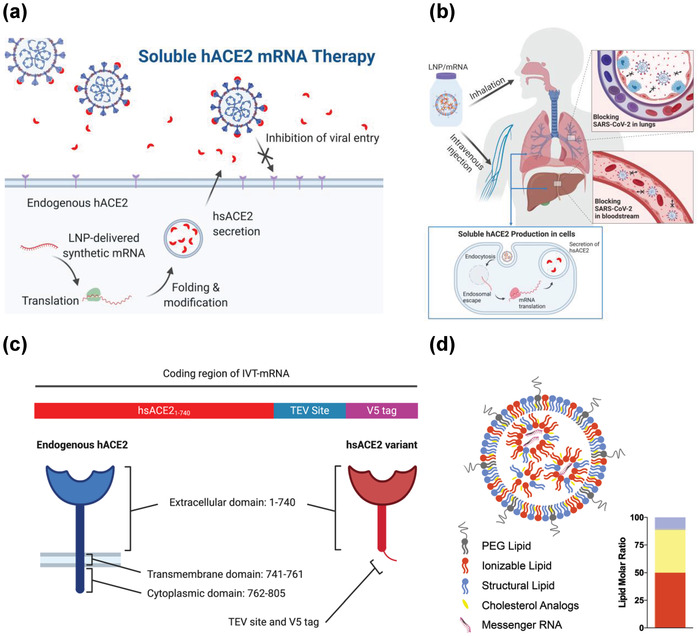
Design of mRNA‐based nanotherapeutic to treat SARS‐CoV‐2 infection. a) A rationale for soluble ACE2 mRNA therapeutic in treating SARS‐CoV‐2 infection. LNP‐delivered synthetic mRNA generates human soluble ACE2 (hsACE2) protein secreted into the extracellular compartment, where it binds receptor binding domain of the spike protein of the SARS‐CoV‐2 and prevents viral entry. b) Potential routes of administration for soluble ACE2 mRNA therapeutic. Intravenous administration of LNP/hsACE2 leads to the production of circulating hsACE2 protein from the liver. Inhalation of LNP/hsACE2 leads to the production of mucosal hsACE2 protein in the lungs. The resulting hsACE2 protein blocks the virus from entering cells. c) A schematic illustration of in vitro‐transcribed (IVT) mRNA encoding extracellular domain of hACE2 protein (red), TEV site (blue), and V5 tag (purple) (top). Endogenous hACE2 consists of extracellular, transmembrane, and cytoplasmic domains (bottom left). The hsACE2 variant contains the extracellular domain of hACE2, a TEV site, and V5‐epitope tag at C‐terminus (bottom right). d) A schematic representation of LNP encapsulating mRNA. Inset, the molar ratio of each lipid in the LNP. Orange; ionizable lipid, Yellow; cholesterol analogs, Blue; structural lipid, Gray; PEG lipid.

To overcome these challenges, we used lipid nanoparticles (LNP) to deliver in vitro‐transcribed messenger RNA (IVT mRNA) for rapid expression of hsACE2 (**Figure** **1**a–c). This strategy aims fast clearance of the captured virus while maintaining hsACE2 levels that can surveil circulation, clear the virus, and rescue the disrupted RAAS system. mRNA‐based therapy is attractive since mRNA synthesis became fast, affordable, and scalable,^[^ ^19^ ^]^ which enabled the rapid development of mRNA vaccines for COVID‐19.^[^ ^20^, ^21^ ^]^ LNP‐delivered mRNA provides a transient yet high protein expression with intrinsic folding and post‐translational modifications but without the risk of insertional mutagenesis.^[^ ^22^, ^23^ ^]^ It can be repeatedly administered to sustain protein production until the infection subsides, unlike virus‐based gene therapy. Moreover, the cessation of the mRNA treatment allows for clearance of hsACE2 within days, mitigating any adverse effects. Here, we hypothesized that the expression of hsACE2 will prevent SARS‐CoV‐2 from binding to cell surface receptors and block its entry (Figure 1a,b). To achieve this, we designed IVT mRNA to encode the first 740 amino acids of hACE2 with a cleavable V5‐epitope tag at the C‐terminus (Figure 1c and Figure S1, Supporting Information). This proof of concept study demonstrated that our mRNA‐based strategy produces hsACE2 that effectively inhibits SARS‐CoV‐2 and its pseudoviruses from infecting host cells, opening its feasibility for the treatment of SARS‐CoV‐2 infection, alone or in combination with other effective antiviral approaches.

## 2 Results

**Figure 2:**
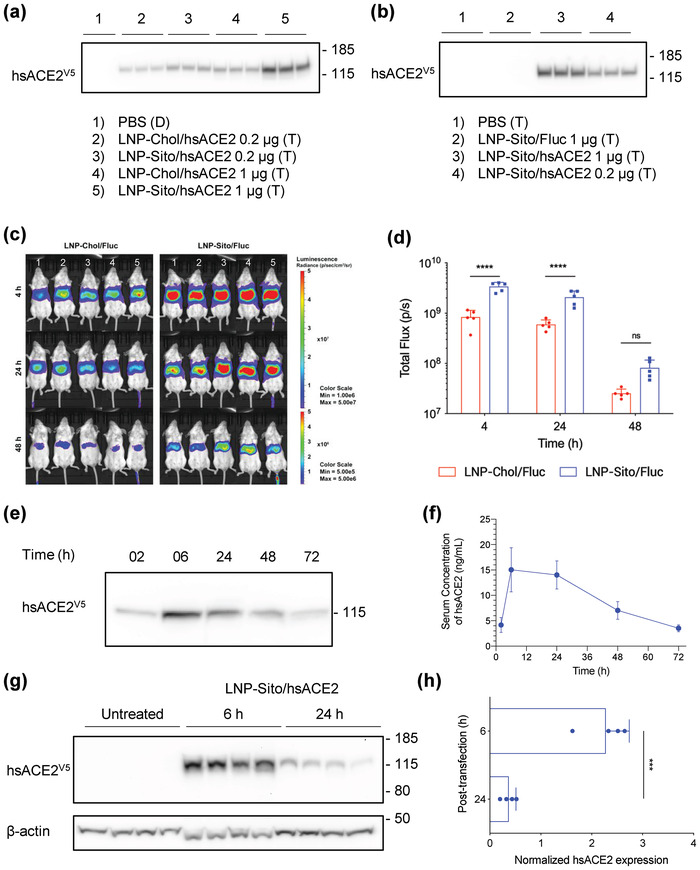
*hsACE2* mRNA transfection rapidly produces the circulatory hsACE2 protein by intravenous administration of lipid nanoparticles. a,b) Western blot of cell‐free conditioned media from a) 293T and b) Hep G2 cell culture after mRNA transfection by various LNP. Treatment and mRNA dose are described under each blot (duplicate (D): *n* = 2, triplicate (T): *n* = 3). c) In vivo bioluminescent images of BALB/c mice after treatment of 0.05 mg kg^−1^ mRNA delivered through IV injection of LNP‐Chol/Fluc or LNP‐Sito/Fluc (*n* = 5). Labels of the mice are found in the numbers on the image. d) Quantification of bioluminescent signals from IVIS images. The region of interest was kept constant in all images (*n* = 5). Statistical analysis was performed using Two‐way repeated‐measures ANOVA with Sidak's multiple comparison tests. ****p < 0.0001; ns (not significant). All data were expressed as the mean ± S.D. e) A representative image of Western blot with mouse sera collected with predetermined time intervals after an IV injection of 0.15 mg kg^−1^ LNP‐Sito/hsACE2. f) Expression kinetics of the circulatory hsACE2 protein in mouse sera after IV injection of LNP‐Sito/hsACE2 (*n* = 5). All data were expressed as the mean ± S.D. g) Western blot of liver homogenates collected from BALB/c mice after IV injection of 0.15 mg kg^−1^ LNP‐Sito/hsACE2. h) Expression of hsACE2 protein in mouse liver homogenates after IV injection of LNP‐Sito/hsACE2 2 (*n* = 4). Densitometric analysis of hsACE2 protein expression normalized to *β*‐actin levels. Statistical analysis was performed using an unpaired *t*‐test. ***p < 0.001. All data were expressed as the mean ± S.D.

To confirm whether the designed IVT mRNA produces hsACE2 protein after transfection, *hsACE2* mRNA was delivered to 293T cells using lipofectamine 3000. We detected the presence of hsACE2 protein in cell‐free conditioned media and cell lysates from *hsACE2* mRNA transfected cells by Western blot but not in PBS‐treated controls (Figure S2a,b, Supporting Information). Efficient intracellular delivery of mRNA, especially in vivo, requires a potent delivery vector. Conventional LNP is composed of four lipids: 1) ionizable lipid, 2) PEG lipid, 3) cholesterol, and 4) structural lipid (Figure 1d). Recently, we discovered a simple substitution of cholesterol to *β*‐sitosterol within LNP formulations can boost intracellular delivery of mRNA by facilitating the endosomal escape of the nanoparticles.^[^ ^24^, ^25^, ^26^ ^]^ Thus, we compared the *β*‐sitosterol LNP (LNP‐Sito: containing *β*‐sitosterol) with the cholesterol LNP (LNP‐Chol: containing cholesterol) as the delivery vector for *hsACE2* mRNA. Both LNP‐Chol and LNP‐Sito encapsulating *hsACE2* mRNA (LNP‐Chol/hsACE2 and LNP‐Sito/hsACE2) exhibited comparable characteristics in terms of hydrodynamic sizes (≈ 80 nm), polydispersity (PDI ≈ 0.08), and mRNA encapsulation (above 98%) (Figure S2c,d, Supporting Information). In addition, we found that LNP‐Sito encapsulating firefly luciferase (*Fluc*) mRNA (LNP‐Sito/Fluc) produced significantly higher luciferase expressions than LNP‐Chol/Fluc (*p* < 0.01) with a dose‐dependent manner in 293T cells without decreasing cell viability, indicating improved transfection efficiency (Figure S3a,b, Supporting Information). In *hsACE2* mRNA delivery, LNP‐Sito/hsACE2 elicited substantially greater production of hsACE2 protein compared to LNP‐Chol/hsACE2 in cell‐free conditioned media from 293T culture. In the cell‐free conditioned media from 293T cells treated with LNP‐Chol/Fluc and LNP‐Sito/Fluc, no expression of hsACE2 was detected in Western blot (Figure S3c, Supporting Information). The improved hsACE2 expression by LNP‐Sito/hsACE2 was confirmed again in the analysis of cell lysates (5‐fold higher expression, *p* < 0.0001) (Figure S3d,e, Supporting Information). Additionally, the production of hsACE2 protein was dependent on the mRNA dose given (**Figure** **2**a and Figure S3f, Supporting Information).

Accumulating evidence suggests that invasion of SARS‐CoV‐2 in ACE2‐expressing airway epithelial cells is followed by infection of endothelial cells, leading to endotheliitis.^[^ ^27^, ^28^ ^]^ Vascular leakage caused by damaged endothelial cells provides the virus with a putative gateway to the circulatory system and other ACE2‐expressing organs.^[^ ^28^ ^]^ Therefore, blockade of the spread of the virus from the blood circulation to peripheral organs is likely to prevent multisystem organ failure. For these reasons, we evaluated in vivo delivery of LNP‐Sito/hsACE2 for production, secretion, and blood circulation of hsACE2 protein. Intravenously administered LNP tend to transfect hepatocytes owing to interactions between LNP and apolipoprotein E.^[^ ^29^ ^]^ Thus, we expected the liver would serve as a factory for protein production upon *hsACE2* mRNA transfection, as shown in other studies for secretory proteins.^[^ ^30^, ^31^ ^]^ To demonstrate this in vitro, the human liver cell line, Hep G2, was transfected with LNP. LNP‐Sito/Fluc yielded more luciferase expression than LNP‐Chol/Fluc in Hep G2 cells (Figure S4a,b, Supporting Information). As was the case with 293T cells, hsACE2 protein was found within the cell‐free conditioned media and lysates harvested from the transfected Hep G2 cells in a dose‐dependent manner (Figure 2b and Figure S4c–e, Supporting Information). Interestingly, in the Hep G2 cell lysates, two discrete hsACE2 bands were observed (Figure S4c, Supporting Information). We assume that the band at ≈125 kDa represented the fully glycosylated form, and the band at 100 kDa meant the pre or partially glycosylated form (Figure S4c, Supporting Information). However, only the fully glycosylated form was detected in the cell‐free media (Figure 2b), suggesting the glycosylation of hsACE2 protein is before secretion. We then examined whether the LNP‐Sito leads to improved protein production in vivo. It was shown that LNP‐Sito/Fluc induced strong bioluminescent signals in the livers of BALB/c mice after intravenous injection of LNP at 4 h post‐injection, which decreased with time (Figure 2c). LNP‐Sito/Fluc exhibited a 3‐fold increase in luciferase expression compared to LNP‐Chol/Fluc at all time points (4–48 h) (Figure 2d). Based on these results, we used LNP‐Sito as an optimized formulation for delivering mRNA in the remaining studies. We injected LNP‐Sito/hsACE2 in BALB/c mice and collected mouse sera up to 72 h post‐administration with predetermined time intervals. Notably, hsACE2 appeared in the mouse sera as early as 2 h postinjection (Figure 2e,f and Figure S5a,b, Supporting Information). This rapid generation of hsACE2 from the liver can be helpful to neutralize SARS‐CoV‐2 promptly at a stage of systemic spread. We found that hsACE2 was detected at the highest level at 6 h post‐injection and gradually declined afterward. We detected circulating hsACE2 even 72 h after a single injection (Figure 2e,f and Figure S5a,b, Supporting Information). The expression of hsACE2 protein in the liver homogenates was time‐dependent, showing a greater expression of the protein after 6 h than 24 h post‐administration (*p* < 0.001) (Figure 2g,h). Unlike the cell lysates of Hep G2, the mouse liver homogenates showed a single band at ≈125 kDa. After 7 days, hsACE2 was eliminated from the blood circulation (data not shown).

**Figure 3:**
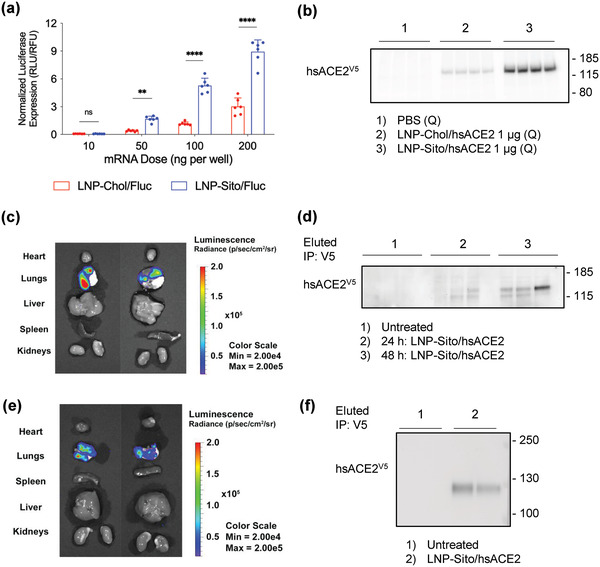
LNP‐delivered *hsACE2* mRNA produced the mucosal hsACE2 protein in the lungs. a) In vitro mRNA transfection of Calu‐3 by treatment of LNP‐Chol/Fluc or LNP‐Sito/Fluc for 48 h at various mRNA doses (*n* = 6). All data were expressed as the mean ± S.D. Statistical analysis was performed using two‐way ANOVA with Sidak's multiple comparison tests. ****p < 0.0001; **p < 0.01; ns (not significant). b) Western blot of cell‐free media of Calu‐3 cell culture after *hsACE2* mRNA transfection using LNP. Treatment and mRNA dose are described under each blot (Q: *n* = 4). c,d) Bioluminescent images of BALB/c mice at 48 h after mRNA treatment by intratracheal instillation (0.5 mg kg^−1^, LNP‐Sito/Fluc). c) In vivo and d) *ex vivo* images of luciferase expression. e) Western blot of hsACE2 protein in the bronchoalveolar lavage fluid (BALF). BALB/c mice were untreated or transfected (0.75 mg kg^−1^, LNP‐Sito/hsACE2) by intratracheal instillation, and the BALF was harvested after 24 or 48 h post‐administration (*n* = 3). For enrichment of the hsACE2 protein, BALF was subjected to immunoprecipitation using an anti‐V5 antibody prior to Western blot. f) Bioluminescent images of organs from BALB/c mice at 24 h after inhalation of 100 µg *Nluc* mRNA delivered by nebulized LNP‐Sito. g) Western blot of hsACE2 protein in the BALF from mice that inhaled LNP‐Sito/hsACE2 at 220 µg mRNA per day for two consecutive days (440 µg of *hsACE2* mRNA in total).

The airway and lungs are the first target organs where the virus attacks and are highly vulnerable tissues due to their high levels of hACE2 expression.^[^ ^11^ ^]^ Having hsACE2 protein as a decoy on the airway epithelium could mitigate viral infection at the early stages of disease progression. Therefore, we explored the ability of LNP to produce mucosal hsACE2. Consistently, LNP‐Sito/Fluc exerted significantly greater levels of transfection than LNP‐Chol/Fluc in Calu‐3, a human lung epithelial cell line (**Figure** **3**a and Figure S6a, Supporting Information). Similarly, LNP‐Sito/hsACE2 showed substantially higher expression of hsACE2 protein than LNP‐Chol/hsACE2 in Western blot (Figure 3b and Figure S6b–d, Supporting Information). To deliver LNP to the mouse lungs locally, we first tried intratracheal instillation as the route of administration. Intratracheally administered LNP‐Sito/Fluc transfected the lungs of BALB/c mouse, and the luciferase expression was detected exclusively in the lungs (Figure 3c,d). However, luciferase expression was unevenly distributed in the lungs, which is likely due to the erratic dispersion of the intratracheally instilled nanoparticles. To evaluate the secretion of hsACE2 to the lung mucosa, we collected bronchoalveolar lavage fluid (BALF) samples at 24 and 48 h post‐administration of LNP‐Sito/hsACE2, followed by a pull‐down assay for hsACE2 protein using an anti‐V5 antibody. We confirmed the presence of mucosal hsACE2 in the collected BALF samples by Western blot (Figure 3e,g). These data represent that lung transfection with LNP‐Sito/hsACE2 resulted in the secretion of hsACE2 to the airway mucus. Having confirmed that delivery of LNP‐Sito/hsACE2 to lung epithelia can produce mucosal hsACE2 protein, we tried to deliver LNP/mRNA to mouse lungs by inhalation. Drug administration by inhalation eases the localized delivery of therapeutics to the pulmonary system with a low systemic distribution. Among several options for inhaled therapies, nebulizers are appropriate for administering the drugs in liquid form. Our group recently showed that nebulized LNP can deliver mRNA to the murine lungs through inhalation.^[^ ^32^ ^]^ Likewise, we used a vibrating mesh nebulizer to aerosolize LNP formulations for the lung delivery of mRNA. For in vivo studies, restrained mice were exposed to the nebulized LNP transported through a tube by airflow, enabling the administration to the lungs without intubation (Figure S7a, Supporting Information). With nebulized LNP‐Sito encapsulating *nanoluciferase* mRNA (*Nluc*), we found bioluminescent signals in the lungs 24 h after treatment (Figure 3f). Similar to the intratracheal instillation of LNP, inhaled LNP delivered mRNA to the lungs without transfecting other organs, such as the liver, spleen, heart, and kidney (Figure 3f). However, we found all five lung lobes displayed luminescent signals, suggesting that inhalation‐mediated mRNA delivery provides impartial transfection to the lungs (Figure S7b, Supporting Information). Next, LNP‐Sito/hsACE2 was administered by inhalation to mice for two consecutive days, followed by BALF collection on the third day. In the pull‐down assay of hsACE2, we found the presence of mucosal hsACE2 in the BALF samples collected from mice that inhaled nebulized LNP‐Sito/hsACE2 (Figure 3g), suggesting that hsACE2 mRNA could be given as an inhalable therapy to produce mucosal protein in the lung epithelia.

**Figure 4:**
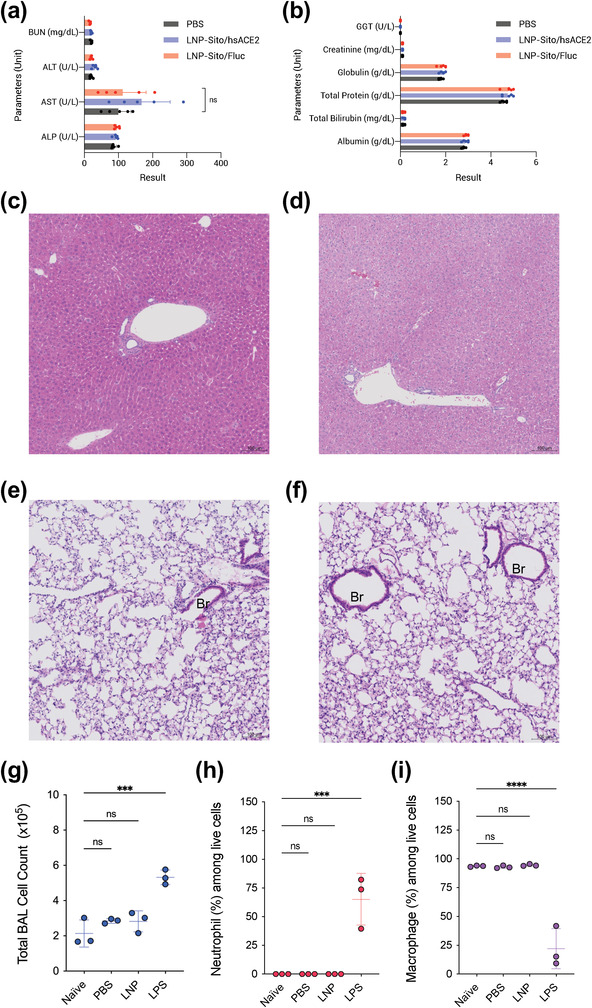
Biochemical and histological analysis of the effects of LNP/mRNA administration. a,b) Blood chemistry results of mouse sera harvested at 24 h after intravenous injection of (black) PBS, (red) LNP‐Sito/Fluc, and (blue) LNP‐Sito/hsACE2 at a dose of 0.5 mg kg^−1^. Data were expressed as the mean ± S.D. (*n* = 5). c,d) Representative H&E stained liver sections of BALB/c mice after intravenous injection of c) PBS or d) LNP‐Sito/hsACE2 at a dose of 0.15 mg kg^−1^. e,f) Representative H&E stained lung sections of BALB/c mice after inhalation of e) PBS or f) LNP‐Sito/Nluc at a dose of 100 µg per mouse. Br; bronchial epithelium. Scale bars represent 100 µm. Magnification: 20×. g–i) Analysis of BAL cells collected from murine lungs that were untreated (naïve) or exposed to inhalation of PBS or LNP‐Sito/Nluc at a dose of 100 µg per mouse, or intranasal instillation of lipopolysaccharide (LPS) at 3 mg kg^−1^ (*n* = 3). g) Total cell counts in BALF. h) Percent of neutrophils among BAL cells i) Percent of macrophages among BAL cells. All data were expressed as the mean ± S.D. Statistical analysis was performed using one‐way ANOVA with Dunnett's multiple comparison tests. ****p < 0.0001; ***p < 0.001; ns (not significant) compared to the naïve group.

Having confirmed that administration of LNP/mRNA via intravenous injection and inhalation is effective in producing hsACE2 proteins, we assessed the safety of the treatments. We did not observe any significant difference in the blood chemistry tests between PBS‐, LNP‐Sito/Fluc, and LNP‐Sito/hsACE2 treated groups at the dose we studied (**Figure** **4**a,b, and Table S1, Supporting Information). Thus, it indicates that intravenous injection of LNP/mRNA does not cause toxicity to the liver and kidney functions at the doses evaluated. We further evaluated the liver tissue damage by histopathology. H&E stained liver sections did not show any difference between PBS and LNP‐Sito/hsACE2 treated mice (Figure 4c,d, and Figure S8a,b and Table S2, Supporting Information), where the livers from both groups were histologically within normal limits. In conjunction with the blood chemistry test results (Table S1, Supporting Information), we concluded that intravenous administration of LNP‐Sito/hsACE2 did not induce liver toxicity in mice at the doses tested. To estimate lung damage associated with LNP/mRNA inhalation, we further measured lactate dehydrogenase (LDH) levels in BALF. Elevated LDH level in BALF is secondary to lung damage in various diseases.^[^ ^33^ ^]^ In BALF samples from PBS or LNP‐Sito/hsACE2 treated lungs, LDH was not detected (Table S3, Supporting Information). We further examined H&E stained lung tissues exposed to LNP‐Sito/Nluc inhalation. We did not observe any apparent increase in immune cell infiltration compared to the PBS control group (Figure 4e,f, and Figure S9a,b, Supporting Information), and the structure of bronchiolar epithelium and alveolar spaces was maintained in both groups (Figure 4e,f, and Figure S9a,b, Supporting Information). To further investigate the pulmonary infiltration of inflammatory cells after LNP/mRNA inhalation, we studied the cellular composition of BAL cells. The BAL cells collected from lipopolysaccharide (LPS) injured lungs were used for a positive control of acute lung injury (Figure 4g–i and Figure S10, Supporting Information). Total cell counts in BALF showed that PBS‐ and LNP‐treated groups had a slight increase (not significant), whereas LPS exposure resulted in a significant increase (*p* < 0.001) in total cell number in BALF (Figure 4g). Next, we looked at neutrophils and macrophages present in BALF samples. Alveolar macrophages are the dominant immune cell in the alveolar compartment of healthy lungs, and neutrophils are the frontline responder marginating to the acute lung inflammation.^[^ ^34^, ^35^ ^]^ We found that neutrophils constituted less than 0.1% in the BAL cells collected from naïve mice and PBS‐ or LNP/mRNA‐exposed mice (Figure 4h). In contrast, LPS exposure significantly increased the neutrophil population to 82% among BAL cells (*p* < 0.001, Figure 4h). In the BAL cells from naïve mice and PBS or LNP/mRNA exposed mice, macrophages appeared to make up more than 90% (Figure 4i). In the BAL cells from LPS‐injured lungs, macrophages accounted for ≈22% (*p* < 0.0001, Figure 4i). These data suggest that LNP/mRNA inhalation did not cause the pulmonary margination of neutrophils. Thus, LNP/mRNA inhalation seems unlikely to cause acute lung injury at our tested dose.

**Figure 5:**
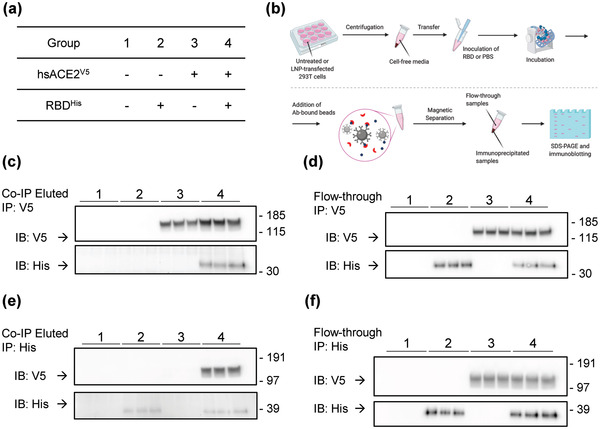
hsACE2 protein binds the receptor‐binding domain of SARS‐CoV‐2 spike protein. a) Cell‐free media from untreated or hsACE2 transfected 293T cell cultures were incubated in the presence or absence of the RBD of the SARS‐CoV‐2 prior to coimmunoprecipitation. + and – define the presence and absence of the treatment, respectively. b) A schematic workflow of coimmunoprecipitation. c,d) Western blot images after coimmunoprecipitation using an anti‐V5 tag antibody. Upper and lower blots were probed using anti‐V5 tag (for hsACE2) and anti‐His tag (for RBD) antibodies, respectively. After immunoprecipitation, c) eluted samples and d) flow‐through samples were analyzed in Western blot. e,f) Western blot images after coimmunoprecipitation using an anti‐His tag antibody. Upper and lower blots were probed using anti‐V5 tag (for hsACE2) and anti‐His tag (for RBD) antibodies, respectively. After immunoprecipitation, e) eluted samples and f) flow‐through samples were analyzed in Western blot.

Next, we evaluated whether there are physical interactions of hsACE2 protein with the RBD of SARS‐CoV‐2. 293T cells were transfected with LNP‐Sito/hsACE2 for 24 h, and untreated cells served as a control. Cell‐free conditioned media were collected and inoculated with either PBS or the recombinant His‐tagged RBD (**Figure** **5**a,b). Coimmunoprecipitation was performed with the samples using the anti‐V5 antibody to capture hsACE2^V5^ (Figure 5b,c). SDS‐PAGE was performed with the immunoprecipitated samples and the flow‐through samples, followed by immunoblotting with anti‐V5 and anti‐His antibodies to confirm the hsACE2 binding to the RBD. Under our experimental conditions, the anti‐V5 antibody precipitated the hsACE2^V5^ from the samples (Figure 5c). The RBD^His^ was detected only in the immunoprecipitated samples that had both hsACE2 and the RBD (Figure 5c), while the samples that contained the RBD^His^ or hsACE2 alone showed no RBD^His^ band in the immunoprecipitated samples (Figure 5c). The unbound RBD^His^ and hsACE2^V5^ were detected in the flow‐through samples, showing the experimental conditions (Figure 5d). We further conducted a reciprocal coimmunoprecipitation, in which the anti‐His antibody was used to capture RBD^His^ (Figure 5e,f). It was observed that the anti‐His antibody coimmunoprecipitated hsACE2^V5^ in the samples that contained both hsACE2^V5^ and RBD^His^ (Figure 5e). hsACE2^V5^ band did not appear in the samples from other groups in immunoblotting. These results demonstrate that hsACE2 protein and the RBD of SARS‐CoV‐2 form a protein complex with a specific and high‐affinity association.

**Figure 6:**
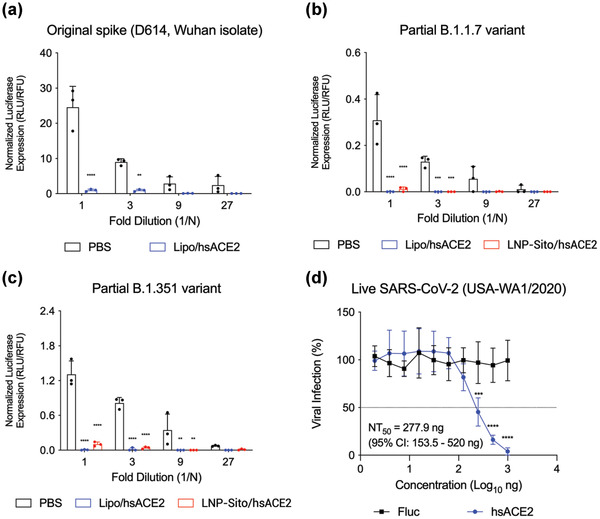
mRNA‐derived hsACE2 protein neutralizes live SARS‐CoV‐2 and the pseudovirus with spike mutations in cells. a–c) Pseudovirus carrying a) the original spike protein (D614) of SARS‐CoV‐2, b) partial B.1.1.7 variant, or c) partial B.1.351 variant in 293T‐hACE2 cells. PBS (black), hsACE2 mRNA‐loaded lipofectamine 3000 (blue), and LNP‐Sito/hsACE2 (red). Pseudovirus were serially diluted for treatment, and normalized luciferase expression was measured (*n* = 3). Statistical analysis was performed using two‐way ANOVA with Sidak's multiple comparison tests. ****p < 0.0001; ***p < 0.001; **p < 0.01 compared to PBS‐treated group. d) Neutralization of SARS‐CoV‐2 infection by mRNA‐derived hsACE2 treatment in Vero E6 cells. Conditioned media derived from *Fluc* mRNA (black) or *hsACE2* mRNA (blue) transfected 293T cells were treated to Vero E6 cells (*n* = 4). All data were expressed as the mean ± S.D. Statistical analysis was performed using two‐way ANOVA with Sidak's multiple comparison tests. ****p < 0.0001; ***p < 0.001; **p < 0.01 compared to *Fluc* mRNA‐treated group.

We further explored the ability of hsACE2 to inhibit the infectivity of SARS‐CoV‐2 using validated neutralization assays with SARS‐CoV‐2 spike pseudotyped lentiviral vectors or natural SARS‐CoV2 infection. For pseudotyping the SARS‐CoV‐2, we utilized *Fluc*‐packaged HIV‐based lentiviral particles containing the spike protein of SARS‐CoV‐2 on them (Figure S11a, Supporting Information). Lentiviral particles with vesicular stomatitis virus G protein (VSV‐G) in place of the spike protein were prepared as a positive control. To investigate hACE2‐dependent infection of the pseudovirus, we created 293T cells stably expressing hACE2 (293T‐hACE2) using a lentiviral vector. The expression of hACE2 in the transduced cells was examined in Western blot, showing the hACE2 band at ≈115 kDa (Figure S11b, Supporting Information). The spike pseudovirus infection was hACE2‐dependent, while VSV‐G pseudovirus infection was hardly affected by hACE2 expression in host cells (Figure S11c,d, Supporting Information). Pseudovirus prepared without envelope protein showed poor infectivity regardless of hACE2 expression in cells (Figure S11e, Supporting Information). After showing hACE2 specificity of the spike pseudovirus, we studied the effects of hsACE2 on the pseudovirus infection. hsACE2‐conditioned media were prepared by transfecting 293T/17 cells with *hsACE2* mRNA. The treatment of hsACE2‐conditioned media led to a great reduction (> 95%) in the transduction of the pseudovirus carrying the original spike protein as compared to the treatment of PBS‐conditioned media (**Figure** **6**a). To further evaluate the inhibitory effects of hsACE2 on spike variants of SARS‐CoV‐2, we produced additional pseudoviruses carrying spike protein mutants as described.^[^ ^36^ ^]^ Partial B.1.1.7 variant had D614G, N501Y mutations, H69/V70 deletion, and 21‐amino‐acid deletion at *C*‐terminus. Partial B.1.351 variant had D614G, N501Y, E484K, and K417N mutations and 21‐amino‐acid deletion at *C*‐terminus. As with the original spike pseudovirus, transduction of both variants was inhibited by treating the hsACE2‐conditioned media (Figure 6b,c). Additionally, LNP‐Sito/hsACE2 treatment produced inhibitory effects comparable to the lipofection of *hsACE2* mRNA against two spike variants. In contrast, the VSV‐G pseudovirus transduction was scarcely inhibited by either treatment (Figure S12, Supporting Information). These data highlight the potent inhibitory effect of hsACE2 treatment on the SARS‐CoV‐2 infection by blocking the spike protein‐mediated viral attachment to hACE2 receptors. Finally, hsACE2 was assessed for neutralizing live SARS‐CoV‐2 USA‐WA1/2020 strain using a plaque reduction microneutralization (PRMNT) assay.^[^ ^37^ ^]^ SARS‐CoV‐2 was treated with the conditioned media before incubating with susceptible Vero E6 cells, followed by an ELISPOT assay to count the number of plaques. This allowed hsACE2 to bind the virus before the virus binds the endogenous ACE2 receptor, therefore blocking the subsequent viral infection. hsACE2 neutralized SARS‐CoV‐2 at a 50% neutralization (NT_50_) of 277.9 ng mL^−1^ (Figure 6d). These results further support that hsACE2 effectively inhibits the SARS‐CoV‐2 in vitro from binding to the cell surface ACE2 receptor or spreading virus progeny from cell to cell.

## 3 Discussion and Conclusion

Despite mass COVID‐19 vaccination, there is an urgent need to develop effective treatment options to end this pandemic. Remdesivir, an antiviral drug approved to treat COVID‐19, has shown encouraging evidence to improve patient recovery.^[^ ^38^ ^]^ Dexamethasone, an anti‐inflammatory steroid repurposed for COVID‐19, has shown to lower mortality in COVID‐19 patients when used in conjunction with respiratory support.^[^ ^39^ ^]^ Several monoclonal antibodies against SARS‐CoV‐2 are also in clinical trials or authorized for emergency use in the United States.^[^ ^40^ ^]^ Nonetheless, the high mutation rate of SARS‐CoV‐2 complicates the development of drugs that treat all variants of concern. This study demonstrates that mRNA‐based nanotherapeutic produces a decoy hsACE2 protein to inhibit the SARS‐CoV‐2 infection. Using a potent LNP formulation (LNP‐Sito), synthetic mRNA was delivered to the cytosol and translated into hsACE2 protein more efficiently than the conventional LNP formulation. Given our previous studies,^[^ ^24^, ^25^, ^26^ ^]^ LNP‐Sito seems to escape better from the endosomal entrapment than LNP‐Chol in vivo as well.^[^ ^26^ ^]^ hsACE2 protein generated from the LNP‐delivered mRNA efficiently bound to the RBD of SARS‐CoV‐2 with a high affinity. It was demonstrated that hsACE2 produced from the delivered mRNA inhibits SARS‐CoV‐2 from infecting susceptible cells. Additionally, the hsACE2 exerted potent neutralizing effects on the pseudovirus carrying the spike protein of the SARS‐CoV‐2 variants, suggesting that hsACE2 would block a range of SARS‐CoV‐2 variants from entering cells. Given that intraperitoneal injection of the recombinant hsACE2‐Fc fusion protein exhibited the prophylactic and therapeutic effects against SARS‐CoV‐2 in mice, this mRNA therapy would be expected to do so in animals.^[^ ^41^ ^]^

Intravenous injection of LNP‐Sito/hsACE2 enabled rapid and sustained expression of the circulating hsACE2 protein in the blood circulation within 2 h, which peaked at 6 h and cleared gradually. Unlike recombinant hsACE2 proteins, the extended availability of mRNA‐derived circulating hsACE2 is due to the continuous production of new proteins from the liver. Given that binding of SARS‐CoV to hACE2 receptors reduces the receptor presentation via SARS‐associated ARDS and soluble ACE2 protects against virus‐mediated lung injury,^[^ ^42^, ^43^ ^]^ sustained expression of hsACE2 during infection could likely facilitate ACE2‐mediated lung protection, reduce the incidence of ARDS by neutralizing SARS‐CoV‐2, and prevent RAAS dysregulation. In fact, Zoufaly et al. showed that the viral load and inflammatory markers in the blood quickly decreased when the intravenous infusion of recombinant hsACE2 (0.4 mg/kg, twice daily for 7 days) was given to COVID‐19 patients.^[^ ^44^ ^]^ Therefore, by binding and thus masking the RBD, hsACE2 mRNA therapy may prevent infection of peripheral organs and the inflammatory responses that cause multisystem organ failure. In addition, the extended residence of hsACE2 of the mRNA therapy would enhance the patient compliance by decreasing the dosing frequency.

Another use of recombinant hsACE2 is to regulate blood pressure in Angiotensin II‐dependent hypertension.^[^ ^45^ ^]^ Prevalence of hypertension among the elderly in the United States is more than 60%,^[^ ^46^ ^]^ and this age group is also at high risk of COVID‐19.^[^ ^47^ ^]^ In this regard, it is conceivable that the expression of enzymatically active hsACE2 from the mRNA therapy could protect COVID‐19 patients with hypertension from aggravation of cardiovascular diseases and viral infection.

While intravenous administration of LNP‐Sito/hsACE2 provides systemic protection against the virus, delivery of hsACE2 mRNA to the respiratory system can be acquired by transfecting lung epithelia. Inhalation and intratracheal instillation of LNP‐Sito/hsACE2 led to the lung transfection and secretion of hsACE2 protein to the airway mucus, the primary site of the virus infection. Although endotracheal administration allows for local delivery of pharmaceuticals to the human lungs, it distributes drugs primarily to the proximal airways and requires invasive procedures, often with mechanical ventilation.^[^ ^48^ ^]^ In contrast, inhalational mRNA therapy provides selective lung transfection with homogenous deposition in a noninvasive manner. It could be useful for treating patients who might not be offered ventilation. In addition, inhalable LNP formulations can effectively deliver mRNA therapeutics to the lungs not only for COVID‐19 but also for other lung diseases that need protein replacement, such as cystic fibrosis.^[^ ^32^ ^]^

Our studies are currently limited to the neutralization effects of the hsACE2 protein against the USA‐WA1/2020 strain of SARS‐CoV‐2 in cells. Although the hsACE2 protein is expected to inhibit other variants from binding to hACE2 receptors,^[^ ^49^ ^]^ the affinity of the hsACE2 protein to other variants could be different, which could affect the virus inhibition. Whether *hsACE2* mRNA treatment could be given with other antiviral drugs might be of interest to treat COVID‐19. Especially for the antiviral drugs that provide a modest efficacy, the synergistic combinations might be a relatively low‐risk treatment option to mitigate COVID‐19. For example, Monteil et al. reported that combining recombinant hsACE2 and Remdesivir produces additive protection against SARS‐CoV‐2 at sub‐toxic concentrations.^[^ ^50^ ^]^ Additional studies that answer these questions would enable the evaluation of the clinical utilities of this therapy. Furthermore, LNP formulations can be optimized for nebulization to enhance the hsACE2 expression upon the mRNA delivery to the lungs. It is known that shear force applied to LNP during nebulization results in dissociation or aggregation of the nanoparticles.^[^ ^51^, ^52^ ^]^ These physicochemical changes of the nanoparticles often lead to the loss of the encapsulated mRNA, which significantly diminishes the therapeutic efficacy and potentially causes adverse effects. Recent studies showed that modulating PEG molarity in LNPs can protect the physicochemical properties during nebulization.^[^ ^52^, ^53^ ^]^ We also reported that combining high PEG molarity and *β*‐sitosterol helped LNPs retain particle properties while providing enhanced pulmonary transfection and mucosal diffusivity.^[^ ^32^ ^]^ Although nebulized LNP‐Sito effectively delivered mRNA to the lungs in this study, new LNP formulations resistant to shear force will enable more efficient mRNA treatment to the lungs. Furthermore, given that the current LNP formulations developed for mRNA vaccines can be immunogenic to the pulmonary system,^[^ ^54^ ^]^ nonirritant formulations are highly necessary to avoid undesirable immune responses during treatment. With the LNP formulations developed for nebulization and safe inhalation, an appropriate and effective dosage regimen could be determined. Potential clinical outcomes of this mRNA therapy would be accurately measured afterward.

mRNA‐based vaccines developed with unprecedented speed have shown to be effective against SARS‐CoV‐2 and its variants.^[^ ^21^, ^55^, ^56^, ^57^ ^]^ mRNA‐based nanotherapeutics that produce hsACE2 or neutralizing antibodies combined with antiviral therapies can emerge as treatment options against SARS‐CoV‐2 infection. Furthermore, it can develop treatments based on high‐affinity hsACE2 variants that bind the RBD of SARS‐CoV‐2 or other hACE2‐exploiting coronaviruses that may spill in the future. We hope this study brings to light the potential of mRNA‐based therapeutics to treat patients suffering from COVID‐19.

## 4 Experimental Section

### Materials

*Fluc* mRNA and *hsACE2* mRNA were purchased from TriLink Biotechnologies (CA, USA). Uridine of *Fluc* mRNA was fully substituted with 5‐methoxyuridine, and uridine and cytidine of hsACE2 mRNA were fully replaced with pseudouridine and 5‐methyl‐cytidine, respectively. Cholesterol and *β*‐sitosterol were purchased from Sigma‐Aldrich. DMG‐PEG_2K_ was bought from NOF America. DLin‐MC3‐DMA and DSPC were obtained from BioFine International Inc. and Avanti Polar Lipids Inc., respectively.

### LNP Formulation and Characterization

LNP, consisting of Dlin‐MC3‐DMA, Cholesterol or *β*‐sitosterol, DMG‐PEG_2K_, DSPC, and mRNA, were prepared using microfluidic mixing as described previously.^[^ ^51^ ^]^ Briefly, mRNA was diluted in sterile 50 mM citrate buffer, and lipid components were prepared in 100% ethanol at a 50:38.5:1.5:10 molar ratio. The lipid and mRNA solutions were mixed using the NanoAssemblr Benchtop (Precision Nanosystems, Inc.) at a 1:3 ratio, followed by overnight dialysis against sterile PBS using a Slide‐A‐Lyzer G2 cassette with 10 000 Da molecular‐weight‐cut‐off (MWCO) (Thermo Fisher Scientific). Dialyzed LNP solutions were concentrated using Amicon Ultra centrifugal filter units with a 10 000 Da MWCO (Millipore). Hydrodynamic size and PDI of the LNP were measured in dynamic light scattering using the Zetasizer Nano ZSP (Malvern Instruments, UK). mRNA encapsulation was assayed using a Quant‐iT RiboGreen RNA Assay kit (Thermo Fisher Scientific) and a multimode microplate reader (Tecan Trading AG).

### Cell Culture

Human 293T, Calu‐3, and Hep G2 cell lines were kindly gifted by Prof. Sadik Esener (OHSU), Prof. Kelvin MacDonald (OHSU), and Prof. Conroy Sun (OSU), respectively. Vero E6 cells were purchased from BEI Resources (NR‐596). The 293T/17 cell line was purchased from ATCC (CRL‐11268). Human 293T, 293T/17, and Hep G2 cells were cultured in DMEM supplemented with 10% heat‐inactivated FBS and 1% penicillin/streptomycin. Calu‐3 cells were cultured in MEM supplemented with 10% heat‐inactivated FBS, 1% penicillin/streptomycin, nonessential amino acids, and sodium pyruvate. Vero E6 cells were cultured in DMEM with supplements as described in the text.

### In Vitro Transcription (IVT) of mRNA

A linearized plasmid containing nanoluciferase (*Nluc*) under the T7 promoter was used as a template for in vitro transcription of *Nluc* mRNA. *Nluc* mRNA was synthesized using the HiScribe T7 High Yield RNA Synthesis Kit (New England Biolabs Inc.) and CleanCap Reagent AG (TriLink Biotechnologies) according to the manufacturer's instructions. Synthesized mRNA was purified using the Monarch RNA Cleanup Kit (New England Biolabs) and stored at −80 °C.

### In Vitro *Fluc* mRNA Transfection Assay

For in vitro *Fluc* mRNA transfection assays, cells were seeded on a white 96 well plate at 4 × 10^3^ cells per well for 293T and Hep G2 cells or at 10^4^ cells/well for Calu‐3, followed by overnight incubation for cell attachment. Cells were incubated with nanoparticles encapsulating *Fluc* mRNA and analyzed for cell viability and luciferase activity with the ONE‐Glo+Tox luciferase reporter and cell viability assay kit (Promega) using a multimode microplate reader.

### In Vitro *hsACE2* mRNA Transfection

For in vitro mRNA transfection for hsACE2 production, cells were seeded on a 12‐well plate at 3 × 10^5^ cells per well and allowed to attach overnight. Cells were treated with LNP encapsulating hsACE2 mRNA for 24 h, and culture media were centrifuged at 500 × *g* for 10 min at 4 °C. Cell‐free media was supplemented with a protease and phosphatase inhibitor cocktail (Thermo Fisher Scientific) and used for downstream experiments. Besides culture media, transfected cells were lysed using RIPA buffer containing protease and phosphatase inhibitor cocktail, followed by centrifugation at 16 000 × *g* for 30 min at 4 °C. The supernatant lysate was collected for Western blot.

### Detection of hsACE2 Protein by Western Blot

Production of hsACE2 protein upon transfection was detected by Western blot. In brief, the total protein concentration of the samples was quantified using a Micro BCA protein assay kit (Thermo Fisher Scientific) according to the manufacturer's instructions. Cell‐free supernatants or cell lysates containing 30 µg of total protein were prepared in 1× LDS sample buffer under reducing conditions, denatured at 70 °C for 10 min, and run on 4–12% Bis‐Tris gels or Tris‐glycine gels, followed by dry transfer to PVDF membrane using iBlot 2 Dry Blotting System (Thermo Fisher Scientific). The blots were blocked using 5% skim milk for 1 h at room temperature (RT). The primary antibodies used were: rabbit monoclonal anti‐V5 tag at 1:1000 (Cell Signaling Technology, 13202), rabbit monoclonal anti‐6x‐His tag at 1:1000 (Thermo Fisher Scientific, MA5‐33032), and mouse monoclonal anti‐*β*‐actin at 1:10 000 (R&D Systems, MAB8929). The secondary antibodies used were goat polyclonal anti‐rabbit HRP (Jackson ImmunoResearch, 111‐035‐003) and anti‐mouse HRP (115‐035‐003). For detection and documentation, SuperSignal West Pico Plus Chemiluminescent Substrate and myECL imager (Thermo Fisher Scientific) were used. After chemiluminescent imaging, blots were further stained using GelCode Blue Safe Protein Stain (Thermo Fisher Scientific) according to the manufacturer's instruction.

### Coimmunoprecipitation (Co‐IP) of hsACE2 and SARS‐CoV‐2 Spike RBD

Cell‐free media from untreated or transfected 293T cell culture was prepared. SARS‐CoV‐2 Spike RBD‐His (1 µg, Sino Biological) was inoculated to 400 µL of cell‐free media, followed by overnight incubation at 4 °C with rotation. According to the manufacturer's instructions, subsequent coimmunoprecipitation was conducted using Dynabeads Protein G Immunoprecipitation Kit (Thermo Fisher Scientific). Briefly, cell‐free media inoculated with the spike RBD were incubated with antibody‐bound Dynabeads for 20 min at RT with rotation. The antibodies used for pull‐down were mouse monoclonal anti‐His tag (sc‐8036) or anti‐V5 tag (sc‐81594) antibody (Santa Cruz Biotechnology). Following three washes with PBS, samples were eluted using elution buffer and denatured using LDS sample buffer and reducing agent for Western blot.

### Animals

All animal studies were conducted at Oregon Health and Sciences University and approved by the Institutional Animal Care and Use Committee (IACUC#, IP00001707).

### In Vivo *Fluc* mRNA Transfection via Intravenous Administration

Female BALB/c mice (8–12 weeks) were sedated using isoflurane, and LNP encapsulating *Fluc* mRNA were administered intravenously via tail vein. At predetermined time points post‐administration, 200 µL of d‐luciferin substrate (Thermo Fisher Scientific) was injected intraperitoneally into the mice 10 min prior to bioluminescence imaging (150 mg kg^−1^). Image acquisition and analysis were performed using the IVIS Lumina XRMS and the manufacturer's software (PerkinElmer).

### In Vivo *hsACE2* mRNA Transfection via Intravenous Administration

Female BALB/c mice (8–12 weeks) were sedated using isoflurane, and LNP encapsulating *hsACE2* mRNA were administered to animals via tail vein. At predetermined time points post‐administration, whole blood was collected using cardiac puncture or submandibular bleeding. The collected blood samples were processed to sera using serum‐separating tubes (BD Biosciences). The separated sera were used for downstream experiments. Mouse liver was sterilely harvested, followed by formalin‐fixation or homogenization using a handheld tissue homogenizer.

### Enzyme‐Linked Immunosorbent Assay (ELISA)

Concentrations of hsACE2 in the mouse sera were quantified using a solid‐phase sandwich ELISA for human ACE2 (R&D Systems) according to the manufacturer's protocol. In brief, a high‐binding ELISA plate was coated with 100 µL of a 2 µg mL^−1^ hACE2 capture antibody overnight at RT. Afterward, the wells were blocked with 1% BSA in 1× PBS overnight at 4 °C. After washing with 1× PBS containing 0.05% Tween‐20, 100 µL of the diluted mouse sera were added to wells in triplicate. After 2 h incubation at RT, 100 µL of a 100 ng mL^−1^ detection antibody solution was added to each well, and the plate was sealed and incubated for 2 h at RT. After thorough washing, 100 µL of a 1:1 mixture of hydrogen peroxide and tetramethylbenzidine was added to each well, and the plate was sealed and incubated in the dark for 20 min at RT. Sulfuric acid (1 n, 100 µL) was added to each well to stop the enzymatic reaction. The plate was read at 450 and 540 nm, and readings at 540 nm were subtracted from readings at 450 nm.

### In Vivo mRNA Transfection via Intratracheal Instillation

Intratracheal instillation was performed as described previously.^[^ ^58^ ^]^ Female BALB/c mice (8–12 weeks) were anesthetized using a ketamine/xylazine cocktail. Anesthetized animals were leaned over an intubation stand (Kent Scientific), and their vocal cords were directly visualized using an otoscope with a 2 mm speculum (Welch Allyn). A flexible wire was advanced through the vocal cords to the trachea. Once the wire was located within the trachea, a 20 G catheter was passed over the wire, and the wire was removed. A gas‐tight syringe with a 22 G blunt needle (Hamilton) was filled with LNP/mRNA for administration. The syringe was inserted through the catheter, and LNP/mRNA was administered to the lungs, followed by 100 µL of air to distribute the LNP solution throughout the lungs.

### In Vivo mRNA Transfection via Inhalation

Inhalation‐mediated mRNA delivery was conducted using a vibrating mesh nebulizer (Aerogen) connected to a plastic spacer and an animal holder (Kent Scientific). LNP encapsulating mRNA were prepared at 0.5 mg mL and added to the nebulizer unit dropwise. LNP solution was added again after the spacer did not contain any visible aerosol. For *Nluc* mRNA delivery, a single dose of 100 µg mRNA encapsulated in LNP‐Sito was given per mouse, followed by *ex vivo* imaging using IVIS after 24 h. For hsACE2 mRNA delivery, 220 µg mRNA encapsulated in LNP‐Sito was given per day per mouse for two consecutive days (440 µg mRNA per mouse in total).

### Histopathology

Mouse livers and lungs were kept in formalin for 24 h. In the following days, the tissues were dissected, placed in tissue embedding cassettes, and submerged in 70% ethanol for dehydration. Tissues were paraffin‐embedded, sectioned, mounted on slides, routinely stained with hematoxylin and eosin, and cover‐slipped allowing histopathologic evaluation by the specialist at IDEXX BioAnalytics. Microscopic changes were graded as to severity utilizing a standard grading system whereby 0 = no significant change, 1 = minimal, 2 = mild, 3 = moderate, and 4 = severe. International Harmonization of Nomenclature and Diagnostic (INHAND) Criteria standards are used for evaluation. The use of numerical grades allows a mechanism to calculate a total score lesion score which can be used to assess the prevalence and severity of tissue changes within and between groups. The slides were imaged using Zeiss Axio Scan.Z1 slide scanner (Zeiss), and the images were cropped using the manufacturer's software (Zen 2 blue edition, Zeiss).

### Collection of Bronchoalveolar Lavage Fluid (BALF)

After pulmonary administration, animals were euthanized humanely at an appropriate time post‐administration. The trachea was surgically exposed and intubated with a 20 G catheter. Mouse lung lavage was performed three times with 0.8 mL of prewarm PBS to collect BALF. Collected BALF was centrifuged at 500 x *g* for 10 min at 4 °C. Supernatants were supplemented with a protease and phosphatase inhibitor cocktail and used for downstream experiments. Cells were resuspended in 200 *µ*L of ACK lysing buffer (Lonza, Walkersville, MD) and incubated for 2 min at RT. 1 mL of cold PBS was added to dilute the ACK lysing buffer, followed by centrifugation at 500 x *g* for 10 min at 4 °C. The supernatant was discarded, and the cells were resuspended in 0.3 mL of PBS to measure cell counts using the Countess 3 Automated Cell Counter (Thermo Fisher Scientific) with trypan blue staining. The total cell number in BALF was calculated as (cell concentration of from each BALF sample) × (Volume of each BALF sample).

### Flow Cytometric Analysis of BAL Cells

The collected BAL cells were stained with antibodies to investigate the cellular composition as described.^[^ ^59^ ^]^ A list of antibodies used for flow cytometry is described in Table S4 (Supporting Information). For cell‐surface antigen staining, the BAL cells were incubated with antibodies for 30 min at 4 °C. The BAL cells were also stained with eBioscience Fixable Viability Dye eFluor 780 (Thermo Fisher Scientific) for dead‐cell discrimination. The BAL cells from the mice treated with intranasal instillation of lipopolysaccharide (3 mg kg^−1^, L6529, Sigma‐Aldrich) were used to mimic neutrophilic inflammation observed in acute lung injury as shown previously.^[^ ^59^, ^60^ ^]^ We analyzed 50 000 events of the stained BAL cells using BD LSRFortessa with FACSDiva version 9.0 (BD Biosciences). The data were analyzed using FlowJo software (version 10.6.2, Tree Star). The percentage of neutrophils and macrophages was calculated as [(Neutrophil or Macrophage cell population) ÷ (Cell counts of the live population)] × 100.

### Lactate Dehydrogenase (LDH) Assay

LDH level in the collected BALF samples was assessed by IDEXX BioAnalytics (Columbia, MO). Briefly, LDH activity was determined enzymatically using the reagents designed for the AU680 Chemistry System (Beckman Coulter) according to the manufacturer's instructions.

### Immunoprecipitation of hsACE2 from BALF

Immunoprecipitation of hsACE2 from BALF was conducted using Dynabeads Protein G Immunoprecipitation Kit (Thermo Fisher Scientific) according to the manufacturer's instruction. The collected BALF was incubated with Dynabeads having anti‐V5 tag antibody with rotation. Following three washes with PBS, samples were eluted using elution buffer and denatured using LDS sample buffer and reducing agent for Western blot.

### Plasmids

Lentiviral reporter plasmid pHAGE‐CMV‐Luc2‐IRES‐ZsGreen‐W (BEI Resources, NR‐52516) and helper plasmids pHDM‐Hgpm2 (BEI Resources, NR‐52517), pHDM‐tatb (BEI Resources, NR‐52518), pRC‐CMV‐rev1b (BEI Resources, NR‐52519), and hACE2 containing pHAGE2‐EF1a ACE2 (BEI Resources, NR‐52512) were kindly provided by Jesse D. Bloom.^[^ ^61^ ^]^ pcDNA3.1‐SARS2‐Spike (Addgene, #145032) was a gift from Fang Li.^[^ ^62^ ^]^ pMD2.G containing VSV‐G envelop protein (Addgene, #12259) and pCMVΔR8.2 (Addgene, #12263) were gifts from Didier Trono. HDM_SARS2_Spike_del21_D614G (Addgene, #158762) encoding the SARS‐CoV‐2 spike protein with the D614G mutation and a 21‐amino‐acid deletion at the C‐terminus was a gift from Jesse Bloom. Plasmids containing partial variants of spike protein were generated by site‐directed mutagenesis.

### Generation of 293T‐hACE2

In order to create 293T/17 cells overexpressing hACE2, the hACE2 gene was transduced to 293T/17 cells using a lentiviral vector. To produce the lentivirus packaging hACE2 gene, pCMVΔR8.2, pMD2.G, and pHAGE2‐EF1aInt‐ACE2‐WT were treated to 293T/17 cells using lipofectamine 2000. After 4 h, the cells were replenished with the fresh growth media. After 48 h, the lentiviral particles were collected, filtered, and used immediately to transduce 293T/17 cells. After 48 h transduction, the cells were harvested, passaged with the growth media, and referred to as 293T‐hACE2. For examining the expression of hACE2 after transduction, the cell lysates were processed to perform Western blot analysis as described above. To probe hACE2, anti‐ACE2 antibody (Santa Cruz Biotechnology, sc‐390851) and anti‐mouse HRP were used as the primary and secondary antibodies at 1:200 and 1:2000, respectively.

### Production of Pseudovirus Particles

293T/17 cells were seeded in a T‐75 flask at 5 × 10^4^ cells/flask and grown for 18 h. Cells were cotransfected with 7.8 µg of the lentiviral reporter, 1.7 µg of each helper plasmids, and either 7.8 µg of pcDNA3.1‐SARS2‐Spike (wild‐type spike or its variants), 2.5 µg of pMD2.G (VSV‐G), or no plasmid (no envelope) using lipofectamine 3000 as instructed by the manufacturer. After 48 h, pseudoviruses were collected, filtered, aliquoted into single‐use vials, and stored at ‐80°C.

### Production of Pseudovirus Variants

Pseudovirus carrying spike variants were generated to incorporate key mutations in the B.1.1.7 and B.1.351 variants.^[^ ^36^ ^]^ Partial B.1.1.7 variant incorporated deletions of amino acids 69 and 70 and a point mutation N501Y. Partial B.1.351 variant incorporated point mutations N501Y, E484K, and K417N. Briefly, the sequence of the spike‐D614G plasmid was changed using a site‐directed mutagenesis kit (New England Biolabs) according to the manufacturer's protocol with minor modifications. All primers and PCR conditions for mutagenesis were determined using NEBaseChanger software (New England Biolabs).

### Titration of Pseudovirus Particles

293T‐hACE2 cells were seeded at 10^4^ cells per well in white, 96‐well plates and grown for 18 h. Cells were transduced in triplicate with a 4‐point, 1:3 serial dilution of the spike pseudoviruses with polybrene at a final concentration of 5 µg mL^−1^. VSV‐G pseudoviruses were diluted 1:40 prior to serial dilution. Polybrene was not included in the VSV‐G pseudovirus‐treated wells. After 48 h, cell viability and luciferase activity were assessed with the ONE‐Glo+Tox luciferase reporter and cell viability assay kit.

### Preparation of Conditioned Media for Virus Neutralization Assays

293T/17 cells were seeded into T‐75 flasks at 5 × 10^4^ cells per flask and grown 18 h. Cells were transfected with 22 µg mRNA or an equivalent volume of PBS using lipofectamine 3000. After incubation for 6 h, cells were washed with PBS and replenished with the complete media. After 24 h, media was harvested, filtered with a 0.45 µm filter, and concentrated in a spin column with Amicon Ultra centrifugal filter units with 10000 Da molecular‐weight cut‐off at 4000 × *g* for 30 min. The concentrated, conditioned media was brought up to 2 mL with serum‐free media and used immediately in the neutralization assays for the pseudovirus or live SARS‐CoV‐2. Aliquots of the conditioned media were assayed for the concentration of hsACE2 using ELISA.

### Pseudovirus Neutralization Assay

For neutralization assay, 293T‐hACE2 cells were seeded into white 96‐well plates at 2 × 10^4^ cells per well and grown for 24 h. Pseudovirus was serially diluted as before. The conditioned media was added to the serial dilutions at a ratio of 2:3 for conditioned media: pseudovirus and incubated at 4 °C for 1 h. Polybrene was added as before. Media was removed from the 96‐well plates, and the cells were transduced as before. After 48 h, cell viability and luciferase activity were assessed with the ONE‐Glo+Tox luciferase reporter and cell viability assay kit.

### SARS‐CoV‐2 Neutralization Assay

hsACE2 was tested for neutralizing SARS‐CoV‐2 USA/WA1/2020 (BEI Resources).^[^ ^37^ ^]^ Briefly, 2‐fold serial dilutions of hsACE2 (starting at 1000 ng mL^−1^) were prepared in a 96‐well plate by adding 50 µL of infection media starting at the second column to the end of the plate. Thereafter, 100 µL of the 1000 ng mL^−1^ of hsACE2 was added to the first column and diluted down the well by transferring 50 µL from the first column to the second, and repeatedly to the other columns down the plate until column 10. The last two columns were left as virus‐only and mock‐only wells. Neutralization assays were conducted in quadruplicates. The conditioned media derived from *Fluc* mRNA treatment served as the negative control for the assay. Then, 100 plaque‐forming units (PFU)/well of SARS‐CoV‐2 were mixed with prediluted hsACE2 in 96 well plates and incubated at 37 °C for 1 h. Vero E6 cells (96‐well plate format, 4 × 10^4^ cells per well, quadruplicates) were infected with the virus‐conditioned media mixture at 37 °C for 1 h, followed by changing media with post‐infection media containing 2% FBS, 1% penicillin/streptomycin/l‐glutamine (PSG), and 1% Avicel. At 24 h post‐infection, infected cells were fixed with 10% neutral buffered formalin for 24 h and were immunostained using anti‐SARS‐CoV‐1/2 nucleocapsid protein monoclonal 1C7C7 antibody (Millipore, Cat# ZMS1075). ELISPOT was used to evaluate and quantify the virus neutralization, and the infectivity was presented in sigmoidal dose‐responsive curves. The virus infection (%) for each concentration is calculated as [(average # of plaques from each treated well) − (average # of plaques from the wells containing no virus)] ÷ [(average # of plaques from the wells containing virus only) − (average # of plaques from the wells containing no virus)] × 100. The 50% neutralization titer (NT_50_) is determined based on a nonlinear regression curve fit analysis over the dilution curve using Prism 8 software (GraphPad, CA, USA).

### Statistical Analysis

Data obtained from this study were used without preprocessing unless otherwise stated. Sample sizes are stated in the corresponding legends. Data are shown as the mean ± S.D. unless otherwise stated. The difference between different groups was analyzed using an unpaired *t*‐test, one‐way ANOVA followed by Dunnett's multiple comparison tests, or two‐way ANOVA followed by Sidak's multiple comparison tests. In all cases, significance was defined as *p* < 0.05. Statistical analysis was carried out using Prism 8 software (GraphPad, CA, USA).

## Conflict of Interest

G.S. is a coinventor in patent application US20200129445A1 that details LNP‐Sito. G.S. is a cofounder of EnterX Bio and RNAvax Bio, and has an advisory role to Saliogen Therapeutics Inc., Rare Air Inc., and Sanofi.

## Supporting information

Supporting Information

## Data Availability

The data that support the findings of this study are available from the corresponding author upon reasonable request.

## References

[1] E. Dong , H. Du , L. Gardner , Lancet Infect. Dis. 2020, 20, 533.32087114

[2] J. Lan , J. Ge , J. Yu , S. Shan , H. Zhou , S. Fan , Q. Zhang , X. Shi , Q. Wang , L. Zhang , X. Wang , Nature 2020, 581, 215. 32225176 10.1038/s41586-020-2180-5

[3] R. Yan , Y. Zhang , Y. Li , L. Xia , Y. Guo , Q. Zhou , Science 2020, 367, 1444. 32132184 10.1126/science.abb2762 PMC7164635

[4] S. Xia , Y. Zhu , M. Liu , Q. Lan , W. Xu , Y. Wu , T. Ying , S. Liu , Z. Shi , S. Jiang , L. Lu , Cell. Mol. Immunol. 2020, 17, 765. 32047258 10.1038/s41423-020-0374-2 PMC7075278

[5] D. Harmer , M. Gilbert , R. Borman , K. L. Clark , FEBS Lett. 2002, 532, 107. 12459472 10.1016/s0014-5793(02)03640-2

[6] I. Hamming , M. Cooper , B. Haagmans , N. Hooper , R. Korstanje , A. Osterhaus , W. Timens , A. Turner , G. Navis , H. van Goor , J. Pathol. 2007, 212, 1. 17464936 10.1002/path.2162 PMC7167724

[7] W. Guan , Z. Ni , Y. Hu , W. Liang , C. Ou , J. He , L. Liu , H. Shan , C. Lei , D. S. C. Hui , B. Du , L. Li , G. Zeng , K.‐Y. Yuen , R. Chen , C. Tang , T. Wang , P. Chen , J. Xiang , S. Li , J. Wang , Z. Liang , Y. Peng , L. Wei , Y. Liu , Y. Hu , P. Peng , J. Wang , J. Liu , Z. Chen , et al., N. Engl. J. Med. 2020, 382, 1708. 32109013 10.1056/NEJMoa2002032 PMC7092819

[8] D. Wang , B. Hu , C. Hu , F. Zhu , X. Liu , J. Zhang , B. Wang , H. Xiang , Z. Cheng , Y. Xiong , Y. Zhao , Y. Li , X. Wang , Z. Peng , JAMA, J. Am. Med. Assoc. 2020, 323, 1061. 10.1001/jama.2020.1585 PMC7042881 32031570

[9] C. Huang , Y. Wang , X. Li , L. Ren , J. Zhao , Y. Hu , L. Zhang , G. Fan , J. Xu , X. Gu , Z. Cheng , T. Yu , J. Xia , Y. Wei , W. Wu , X. Xie , W. Yin , H. Li , M. Liu , Y. Xiao , H. Gao , L. Guo , J. Xie , G. Wang , R. Jiang , Z. Gao , Q. Jin , J. Wang , B. Cao , Lancet 2020, 395, 497. 31986264 10.1016/S0140-6736(20)30183-5 PMC7159299

[10] A. Gupta , M. V. Madhavan , K. Sehgal , N. Nair , S. Mahajan , T. S. Sehrawat , B. Bikdeli , N. Ahluwalia , J. C. Ausiello , E. Y. Wan , D. E. Freedberg , A. J. Kirtane , S. A. Parikh , M. S. Maurer , A. S. Nordvig , D. Accili , J. M. Bathon , S. Mohan , K. A. Bauer , M. B. Leon , H. M. Krumholz , N. Uriel , M. R. Mehra , M. S. V. Elkind , G. W. Stone , A. Schwartz , D. D. Ho , J. P. Bilezikian , D. W. Landry , Nat. Med. 2020, 26, 1017. 32651579 10.1038/s41591-020-0968-3 PMC11972613

[11] H. Zhang , J. M. Penninger , Y. Li , N. Zhong , A. S. Slutsky , Intensive Care Med. 2020, 46, 586. 32125455 10.1007/s00134-020-05985-9 PMC7079879

[12] R. Ameratunga , K. Lehnert , E. Leung , D. Comoletti , R. Snell , S.‐T. Woon , W. Abbott , E. Mears , R. Steele , J. McKee , S. Das , W. Rolleston , M. E. Quiñones‐Mateu , H. Petousis‐Harris , A. Jordan , N. Z. Med. J. 2020, 133, 7. 32438383

[13] A. C. Walls , Y.‐J. Park , M. A. Tortorici , A. Wall , A. T. McGuire , D. Veesler , Cell 2020, 181, 281. 32155444 10.1016/j.cell.2020.02.058 PMC7102599

[14] V. Monteil , H. Kwon , P. Prado , A. Hagelkrüys , R. A. Wimmer , M. Stahl , A. Leopoldi , E. Garreta , C. Hurtado del Pozo , F. Prosper , J. P. Romero , G. Wirnsberger , H. Zhang , A. S. Slutsky , R. Conder , N. Montserrat , A. Mirazimi , J. M. Penninger , Cell 2020, 181, 905. 32333836 10.1016/j.cell.2020.04.004 PMC7181998

[15] M. Haschke , M. Schuster , M. Poglitsch , H. Loibner , M. Salzberg , M. Bruggisser , J. Penninger , S. Krähenbühl , Clin. Pharmacokinet. 2013, 52, 783. 23681967 10.1007/s40262-013-0072-7

[16] C. Lei , K. Qian , T. Li , S. Zhang , W. Fu , M. Ding , S. Hu , Nat. Commun. 2020, 11, 2070. 32332765 10.1038/s41467-020-16048-4 PMC7265355

[17] T. Tada , C. Fan , J. S. Chen , R. Kaur , K. A. Stapleford , H. Gristick , B. M. Dcosta , C. B. Wilen , C. M. Nimigean , N. R. Landau , Cell Rep. 2020, 33, 108528. 33326798 10.1016/j.celrep.2020.108528 PMC7705358

[18] P. Liu , J. Wysocki , T. Souma , M. Ye , V. Ramirez , B. Zhou , L. D. Wilsbacher , S. E. Quaggin , D. Batlle , J. Jin , Kidney Int. 2018, 94, 114. 29691064 10.1016/j.kint.2018.01.029 PMC13662898

[19] B. S. Graham , J. R. Mascola , A. S. Fauci , JAMA, J. Am. Med. Assoc. 2018, 319, 1431. 10.1001/jama.2018.0345 29566112

[20] National Institute of Allergy and Infectious Diseases (NIAID) , Phase I, Open‐Label, Dose‐Ranging Study of the Safety and Immunogenicity of 2019‐NCoV Vaccine (MRNA‐1273) in Healthy Adults, Clinicaltrials.Gov, 2020.

[21] K. S. Corbett , D. K. Edwards , S. R. Leist , O. M. Abiona , S. Boyoglu‐Barnum , R. A. Gillespie , S. Himansu , A. Schäfer , C. T. Ziwawo , A. T. DiPiazza , K. H. Dinnon , S. M. Elbashir , C. A. Shaw , A. Woods , E. J. Fritch , D. R. Martinez , K. W. Bock , M. Minai , B. M. Nagata , G. B. Hutchinson , K. Wu , C. Henry , K. Bahi , D. Garcia‐Dominguez , L. Ma , I. Renzi , W.‐P. Kong , S. D. Schmidt , L. Wang , Y. Zhang , et al., Nature 2020, 586, 567. 32756549 10.1038/s41586-020-2622-0 PMC7581537

[22] H. Yin , R. L. Kanasty , A. A. Eltoukhy , A. J. Vegas , J. R. Dorkin , D. G. Anderson , Nat. Rev. Genet. 2014, 15, 541. 25022906 10.1038/nrg3763

[23] J. Kim , Y. Eygeris , M. Gupta , G. Sahay , Adv. Drug Delivery Rev. 2021, 170, 83. 10.1016/j.addr.2020.12.014 PMC7837307 33400957

[24] Y. Eygeris , S. Patel , A. Jozic , G. Sahay , Nano Lett. 2020, 20, 4543. 32375002 10.1021/acs.nanolett.0c01386

[25] S. Patel , N. Ashwanikumar , E. Robinson , Y. Xia , C. Mihai , J. P. Griffith , S. Hou , A. A. Esposito , T. Ketova , K. Welsher , J. L. Joyal , Ö. Almarsson , G. Sahay , Nat. Commun. 2020, 11, 983. 32080183 10.1038/s41467-020-14527-2 PMC7033178

[26] M. Herrera , J. Kim , Y. Eygeris , A. Jozic , G. Sahay , Biomater. Sci. 2021, 9, 4289. 33586742 10.1039/d0bm01947j PMC8769212

[27] L.‐A. Teuwen , V. Geldhof , A. Pasut , P. Carmeliet , Nat. Rev. Immunol. 2020, 20, 389. 32439870 10.1038/s41577-020-0343-0 PMC7240244

[28] Z. Varga , A. J. Flammer , P. Steiger , M. Haberecker , R. Andermatt , A. S. Zinkernagel , M. R. Mehra , R. A. Schuepbach , F. Ruschitzka , H. Moch , Lancet 2020, 395, 1417. 32325026 10.1016/S0140-6736(20)30937-5 PMC7172722

[29] A. Akinc , W. Querbes , S. De , J. Qin , M. Frank‐Kamenetsky , K. N. Jayaprakash , M. Jayaraman , K. G. Rajeev , W. L. Cantley , J. R. Dorkin , J. S. Butler , L. Qin , T. Racie , A. Sprague , E. Fava , A. Zeigerer , M. J. Hope , M. Zerial , D. W. Sah , K. Fitzgerald , M. A. Tracy , M. Manoharan , V. Koteliansky , A. de Fougerolles , M. A. Maier , Mol. Ther. 2010, 18, 1357. 20461061 10.1038/mt.2010.85 PMC2911264

[30] M. S. D. Kormann , G. Hasenpusch , M. K. Aneja , G. Nica , A. W. Flemmer , S. Herber‐Jonat , M. Huppmann , L. E. Mays , M. Illenyi , A. Schams , M. Griese , I. Bittmann , R. Handgretinger , D. Hartl , J. Rosenecker , C. Rudolph , Nat. Biotechnol. 2011, 29, 154. 21217696 10.1038/nbt.1733

[31] Y. Rybakova , P. S. Kowalski , Y. Huang , J. T. Gonzalez , M. W. Heartlein , F. DeRosa , D. Delcassian , D. G. Anderson , Mol. Ther. 2019, 27, 1415. 31160223 10.1016/j.ymthe.2019.05.012 PMC6698250

[32] J. Kim , A. Jozic , Y. Lin , Y. Eygeris , E. Bloom , X. Tan , C. Acosta , K. D. MacDonald , K. D. Welsher , G. Sahay , ACS Nano 2022, 16, 14792. 36038136 10.1021/acsnano.2c05647 PMC9939008

[33] A. Emad , V. Emad , J Cancer Res Clin Oncol 2008, 134, 489. 17882455 10.1007/s00432-007-0311-0 PMC12161628

[34] B. T. Thompson , R. C. Chambers , K. D. Liu , N. Engl. J. Med. 2017, 377, 562. 28792873 10.1056/NEJMra1608077

[35] R. L. Zemans , M. A. Matthay , Thorax 2017, 72, 1. 27974631 10.1136/thoraxjnl-2016-209170 PMC5889088

[36] K. Wu , A. P. Werner , M. Koch , A. Choi , E. Narayanan , G. B. E. Stewart‐Jones , T. Colpitts , H. Bennett , S. Boyoglu‐Barnum , W. Shi , J. I. Moliva , N. J. Sullivan , B. S. Graham , A. Carfi , K. S. Corbett , R. A. Seder , D. K. Edwards , N. Engl. J. Med. 2021, 384, 1468. 33730471 10.1056/NEJMc2102179 PMC8008744

[37] J.‐G. Park , F. S. Oladunni , K. Chiem , C. Ye , M. Pipenbrink , T. Moran , M. R. Walter , J. Kobie , L. Martinez‐Sobrido , J. Virol. Methods 2021, 287, 113995. 33068703 10.1016/j.jviromet.2020.113995 PMC7554492

[38] J. H. Beigel , K. M. Tomashek , L. E. Dodd , A. K. Mehta , B. S. Zingman , A. C. Kalil , E. Hohmann , H. Y. Chu , A. Luetkemeyer , S. Kline , D. Lopez de Castilla , R. W. Finberg , K. Dierberg , V. Tapson , L. Hsieh , T. F. Patterson , R. Paredes , D. A. Sweeney , W. R. Short , G. Touloumi , D. C. Lye , N. Ohmagari , M. Oh , G. M. Ruiz‐Palacios , T. Benfield , G. Fätkenheuer , M. G. Kortepeter , R. L. Atmar , C. B. Creech , J. Lundgren , et al., N. Engl. J. Med. 2020, 383, 1813. 32445440 10.1056/NEJMoa2007764 PMC7262788

[39] The RECOVERY Collaborative Group , N. Engl. J. Med. 2021, 384, 693. 32678530 10.1056/NEJMoa2021436 PMC7383595

[40] P. C. Taylor , A. C. Adams , M. M. Hufford , I. de la Torre , K. Winthrop , R. L. Gottlieb , Nat. Rev. Immunol. 2021, 21, 382. 33875867 10.1038/s41577-021-00542-x PMC8054133

[41] Z. Zhang , E. Zeng , L. Zhang , W. Wang , Y. Jin , J. Sun , S. Huang , W. Yin , J. Dai , Z. Zhuang , Z. Chen , J. Sun , A. Zhu , F. Li , W. Cao , X. Li , Y. Shi , M. Gan , S. Zhang , P. Wei , J. Huang , N. Zhong , G. Zhong , J. Zhao , Y. Wang , W. Shao , J. Zhao , Cell Discov. 2021, 7, 65. 34385423 10.1038/s41421-021-00302-0 PMC8359631

[42] K. Kuba , Y. Imai , S. Rao , H. Gao , F. Guo , B. Guan , Y. Huan , P. Yang , Y. Zhang , W. Deng , L. Bao , B. Zhang , G. Liu , Z. Wang , M. Chappell , Y. Liu , D. Zheng , A. Leibbrandt , T. Wada , A. S. Slutsky , D. Liu , C. Qin , C. Jiang , J. M. Penninger , Nat. Med. 2005, 11, 875. 16007097 10.1038/nm1267 PMC7095783

[43] Y. Imai , K. Kuba , S. Rao , Y. Huan , F. Guo , B. Guan , P. Yang , R. Sarao , T. Wada , H. Leong‐Poi , M. A. Crackower , A. Fukamizu , C.‐C. Hui , L. Hein , S. Uhlig , A. S. Slutsky , C. Jiang , J. M. Penninger , Nature 2005, 436, 112. 16001071 10.1038/nature03712 PMC7094998

[44] A. Zoufaly , M. Poglitsch , J. H. Aberle , W. Hoepler , T. Seitz , M. Traugott , A. Grieb , E. Pawelka , H. Laferl , C. Wenisch , S. Neuhold , D. Haider , K. Stiasny , A. Bergthaler , E. Puchhammer‐Stoeckl , A. Mirazimi , N. Montserrat , H. Zhang , A. S. Slutsky , J. M. Penninger , Lancet Respir. Med. 2020, 8, 1154. 33131609 10.1016/S2213-2600(20)30418-5 PMC7515587

[45] J. Wysocki , M. Ye , E. Rodriguez , F. R. González‐Pacheco , C. Barrios , K. Evora , M. Schuster , H. Loibner , K. B. Brosnihan , C. M. Ferrario , J. M. Penninger , D. Batlle , Hypertension 2010, 55, 90. 19948988 10.1161/HYPERTENSIONAHA.109.138420 PMC2827767

[46] C. D. Fryar , Y. Ostchega , C. M. Hales , G. Zhang , D. Kruszon‐Moran , NCHS Data Brief 2017, 289, 1. 29155682

[47] CDC COVID‐19 Response Team, CDC COVID‐19 Response Team , S. Bialek , E. Boundy , V. Bowen , N. Chow , A. Cohn , N. Dowling , S. Ellington , R. Gierke , A. Hall , J. MacNeil , P. Patel , G. Peacock , T. Pilishvili , H. Razzaghi , N. Reed , M. Ritchey , E. Sauber‐Schatz , MMWR Morb Mortal Wkly. Rep. 2020, 69, 343. 32214079 10.15585/mmwr.mm6912e2 PMC7725513

[48] A. K. Patel , J. C. Kaczmarek , S. Bose , K. J. Kauffman , F. Mir , M. W. Heartlein , F. DeRosa , R. Langer , D. G. Anderson , Adv. Mater. 2019, 31, 1805116. 10.1002/adma.201805116 PMC7490222 30609147

[49] V. Monteil , B. Eaton , E. Postnikova , M. Murphy , B. Braunsfeld , I. Crozier , F. Kricek , J. Niederhöfer , A. Schwarzböck , H. Breid , S. Devignot , J. Klingström , C. Thålin , M. J. Kellner , W. Christ , S. Havervall , S. Mereiter , S. Knapp , A. Sanchez Jimenez , A. Bugajska‐Schretter , A. Dohnal , C. Ruf , R. Gugenberger , A. Hagelkruys , N. Montserrat , I. Kozieradzki , O. Hasan Ali , J. Stadlmann , M. R. Holbrook , C. Schmaljohn , et al., EMBO Mol. Med. 2022, 14, 15230. 10.15252/emmm.202115230 PMC9350269 35781796

[50] V. Monteil , M. Dyczynski , V. M. Lauschke , H. Kwon , G. Wirnsberger , S. Youhanna , H. Zhang , A. S. Slutsky , C. Hurtado del Pozo , M. Horn , N. Montserrat , J. M. Penninger , A. Mirazimi , EMBO Mol. Med. 2021, 13, 13426. 10.15252/emmm.202013426 PMC7799356 33179852

[51] J. Kim , A. Jozic , G. Sahay , Cell. Mol. Bioeng. 2020, 13, 463. 32837581 10.1007/s12195-020-00619-y PMC7250267

[52] H. Zhang , J. Leal , M. R. Soto , H. D. C. Smyth , D. Ghosh , Pharmaceutics 2020, 12, 1042. 33143328 10.3390/pharmaceutics12111042 PMC7692784

[53] M. P. Lokugamage , D. Vanover , J. Beyersdorf , M. Z. C. Hatit , L. Rotolo , E. S. Echeverri , H. E. Peck , H. Ni , J.‐K. Yoon , Y. Kim , P. J. Santangelo , J. E. Dahlman , Nat. Biomed. Eng. 2021, 5, 1059. 34616046 10.1038/s41551-021-00786-x PMC10197923

[54] S. Ndeupen , Z. Qin , S. Jacobsen , A. Bouteau , H. Estanbouli , B. Z. Igyártó , iScience 2021, 24, 103479. 34841223 10.1016/j.isci.2021.103479 PMC8604799

[55] J. Lopez Bernal , N. Andrews , C. Gower , E. Gallagher , R. Simmons , S. Thelwall , J. Stowe , E. Tessier , N. Groves , G. Dabrera , R. Myers , C. N. J. Campbell , G. Amirthalingam , M. Edmunds , M. Zambon , K. E. Brown , S. Hopkins , M. Chand , M. Ramsay , N. Engl. J. Med. 2021, 385, 585. 34289274 10.1056/NEJMoa2108891 PMC8314739

[56] Y. Liu , J. Liu , H. Xia , X. Zhang , J. Zou , C. R. Fontes‐Garfias , S. C. Weaver , K. A. Swanson , H. Cai , R. Sarkar , W. Chen , M. Cutler , D. Cooper , A. Muik , U. Sahin , K. U. Jansen , X. Xie , P. R. Dormitzer , P.‐Y. Shi , N. Engl. J. Med. 2021, 385, 472. 33979486 10.1056/NEJMc2106083 PMC8133696

[57] J. Liu , Y. Liu , H. Xia , J. Zou , S. C. Weaver , K. A. Swanson , H. Cai , M. Cutler , D. Cooper , A. Muik , K. U. Jansen , U. Sahin , X. Xie , P. R. Dormitzer , P.‐Y. Shi , Nature 2021, 596, 273. 34111888 10.1038/s41586-021-03693-y

[58] M. B. Lawrenz , R. A. Fodah , M. G. Gutierrez , J. Warawa , J. Vis. Exp. 2014, 52261, 10.3791/52261. 25490457 PMC4354010

[59] L. Van Hoecke , E. R. Job , X. Saelens , K. Roose , J Vis Exp 2017, 55398. 28518083 10.3791/55398 PMC5607888

[60] F. Khadangi , A.‐S. Forgues , S. Tremblay‐Pitre , A. Dufour‐Mailhot , C. Henry , M. Boucher , M.‐J. Beaulieu , M. Morissette , L. Fereydoonzad , D. Brunet , A. Robichaud , Y. Bossé , Sci. Rep. 2021, 11, 7777. 33833346 10.1038/s41598-021-87462-x PMC8032690

[61] K. H. D. Crawford , R. Eguia , A. S. Dingens , A. N. Loes , K. D. Malone , C. R. Wolf , H. Y. Chu , M. A. Tortorici , D. Veesler , M. Murphy , D. Pettie , N. P. King , A. B. Balazs , J. D. Bloom , Viruses 2020, 12, 513. 32384820 10.3390/v12050513 PMC7291041

[62] J. Shang , G. Ye , K. Shi , Y. Wan , C. Luo , H. Aihara , Q. Geng , A. Auerbach , F. Li , Nature 2020, 581, 221. 32225175 10.1038/s41586-020-2179-y PMC7328981

