# Supplementary material for: Rapid Generation of Circulating and Mucosal Decoy Human ACE2 using mRNA Nanotherapeutics for the Potential Treatment of SARS‐CoV‐2
Source: Adv Sci (Weinh). 2022 Oct 10;9(35):2202556. doi: 10.1002/advs.202202556 (PMC9762296; doi:10.1002/advs.202202556)
Supplement: Supplementary file 1 — Supporting Information [file ADVS-9-2202556-s001.pdf]

ADVANCED SCIENCE
Open Access

# Supporting Information

for *Adv. Sci.*, DOI 10.1002/advs.202202556

Rapid Generation of Circulating and Mucosal Decoy Human ACE2 using mRNA Nanotherapeutics for the Potential Treatment of SARS-CoV-2

*Jeonghwan Kim, Antony Jozic, Anindit Mukherjee, Dylan Nelson, Kevin Chiem, Md Siddiqur Rahman Khan, Jordi B. Torrelles, Luis Martinez-Sobrido and Gaurav Sahay**

Supporting Information

**Rapid generation of circulating and mucosal decoy human ACE2 using mRNA nanotherapeutics for the potential treatment of SARS-CoV-2**

*Jeonghwan Kim, Antony Jozic, Anindit Mukherjee, Dylan Nelson, Kevin Chiem, Md Siddiqur Rahman Khan, Jordi B. Torrelles, Luis Martinez-Sobrido, Gaurav Sahay*[*]

**Figure S1: mRNA sequence of hsACE2 variant**

aug uca agc ucu ucc ugg cuc cuu cuc agc cuu guu gcu gua acu gcu gcu cag ucc acc auu gag gaa cag gcc aag aca uuu uug gac aag uuu aac cac gaa gcc gaa gac cug uuc uau caa agu uca cuu gcu ucu ugg aau uau aac acc aau auu acu gaa gag aau guc caa aac aug aau aac gcu ggg gac aaa ugg ucu gcc uuu uua aag gaa cag ucc aca cuu gcc caa aug uau cca cua caa gaa auu cag aau cuc aca guc aag cuu cag cug cag gcu cuu cag caa aau ggg ucu uca gug cuc uca gaa gac aag agc aaa cgg uug aac aca auu cua aau aca aug agc acc auc uac agu acu gga aaa guu ugu aac cca gau aau cca caa gaa ugc uua uua cuu gaa cca ggu uug aau gaa aua aug gca aac agu uua gac uac aau gag agg cuc ugg gcu ugg gaa agc ugg aga ucu gag guc ggc aag cag cug agg cca uua uau gaa gag uau gug guc uug aaa aau gag aug gca aga gca aau cau uau gag gac uau ggg gau uau ugg aga gga gac uau gaa gua aau ggg gua gau ggc uau gac uac agc cgc ggc cag uug auu gaa gau gug gaa cau acc uuu gaa gag auu aaa cca uua uau gaa cau cuu cau gcc uau gug agg gca aag uug aug aau gcc uau ccu ucc uau auc agu cca auu gga ugc cuc ccu gcu cau uug cuu ggu gau aug ugg ggu aga uuu ugg aca aau cug uac ucu uug aca guu ccc uuu gga cag aaa cca aac aua gau guu acu gau gca aug gug gac cag gcc ugg gau gca cag aga aua uuc aag gag gcc gag aag uuc uuu gua ucu guu ggu cuu ccu aau aug acu caa gga uuc ugg gaa aau ucc aug cua acg gac cca gga aau guu cag aaa gca guc ugc cau ccc aca gcu ugg gac cug ggg aaa ggc gac uuc agg auc cuu aug ugc aca aag gug aca aug gac gac uuc cug aca gcu cau cau gag aug ggg cau auu cag uau gau aug gca uau gcu gca caa ccu uuu cug cua aga aau gga gcu aau gaa gga uuc cau gaa gcu guu ggg gaa auc aug uca cuu ucu gca gcc aca ccu aag cau uua aaa ucc auu ggu cuu cug uca ccc gau uuu caa gaa gac aau gaa aca gaa aua aac uuc cug cuc aaa caa gca cuc acg auu guu ggg acu cug cca uuu acu uac aug uua gag aag ugg agg ugg aug guc uuu aaa ggg gaa auu ccc aaa gac cag ugg aug aaa aag ugg ugg gag aug aag cga gag aua guu ggg gug gug gaa ccu gug ccc cau gau gaa aca uac ugu gac ccc gca ucu cug uuc cau guu ucu aau gau uac uca uuc auu cga uau uac aca agg acc cuu uac caa uuc cag uuu caa gaa gca cuu ugu caa gca gcu aaa cau gaa ggc ccu cug cac aaa ugu gac auc uca aac ucu aca gaa gcu gga cag aaa cug uuc aau aug cug agg cuu gga aaa uca gaa ccc ugg acc cua gca uug gaa aau guu gua gga gca aag aac aug aau gua agg cca cug cuc aac uac uuu gag ccc uua uuu acc ugg cug aaa gac cag aac aag aau ucu uuu gug gga ugg agu acc gac ugg agu cca uau gca gac caa agc auc aaa gug agg aua agc cua aaa uca gcu cuu gga gau aga gca uau gaa ugg aac gac aau gaa aug uac cug uuc cga uca ucu guu gca uau gcu aug agg cag uac uuu uua aaa gua aaa aau cag aug auu cuu uuu ggg gag gag gau gug cga gug gcu aau uug aaa cca aga auc ucc uuu aau uuc uuu guc acu gca ccu aaa aau gug ucu gau auc auu ccu aga acu gaa guu gaa aag gcc auc agg aug ucc cgg agc cgu auc aau gau gcu uuc cgu cug aau gac aac agc cua gag uuu cug ggg aua cag cca aca cuu gga ccu ccu aac cag ccc ccu guu ucc gag aac uug uac uuc caa ucc ggu aag ccu auc ccu aac ccu cuc cuc ggu cuc gau ucu acg uaa

Start/Stop codon
hsACE2 sequence
TEV site
V5 tag

Note: Green bases are start (aug) and stop (uaa) codons. Red sequence represents the first 740 amino acids of the human ACE2 (hACE2) protein. Blue bases represent the TEV (Tobacco Etch Virus) protease site that can be used as a cleavage site for the removal of the C-terminal V5 epitope tag. Purple bases represent the V5 epitope tag.

**Figure S2: mRNA derived hsACE2 protein expression and characterization of LNPs encapsulating the mRNA**

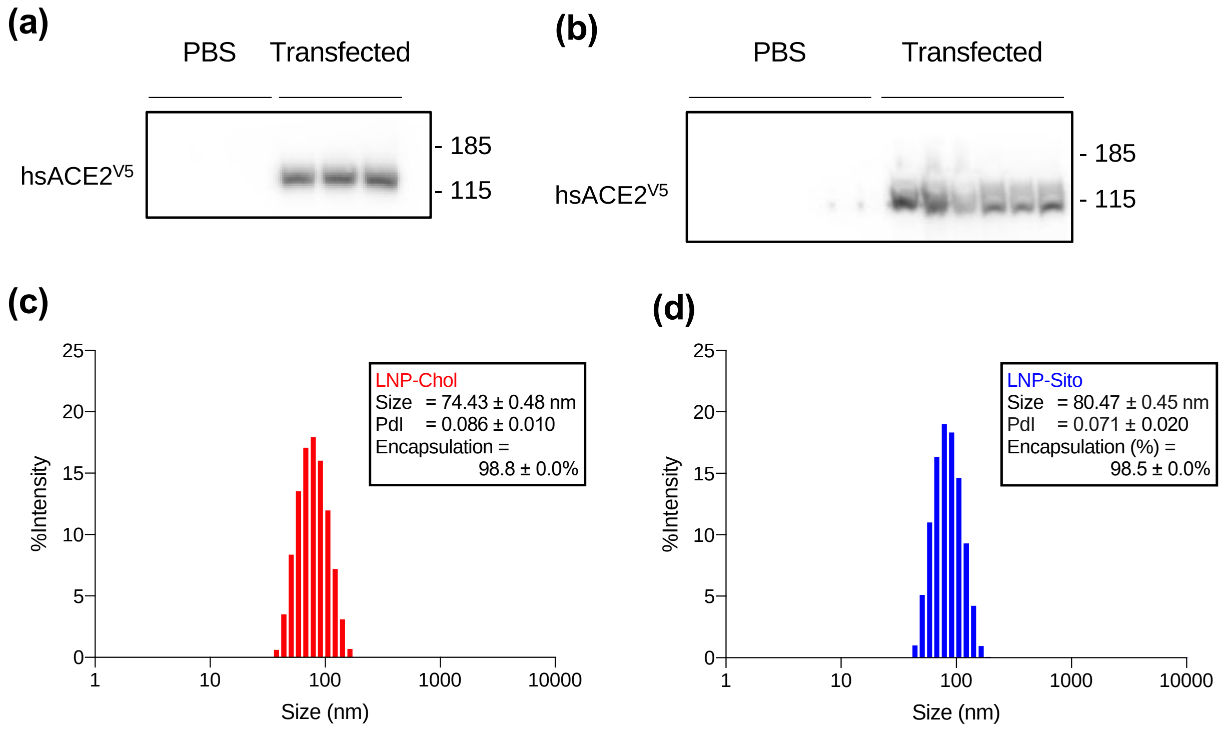


(**a,b**) Western blot of (**a**) cell-free media and (**b**) cell lysates derived from 293T cell culture that was transfected with hsACE2 mRNA using lipofectamine 3000 for 24 hours. Cell-free media and cell lysates of PBS-treated cells were used as internal controls. (**c,d**) Representative data of size distribution (nm) and RNA encapsulation (%) of (**c**) LNP-Chol/hsACE2 and (**d**) LNP-Sito/hsACE2 that were used in the study.

## Figure S3: Nanoparticle-delivered mRNA transfection in 293T cells

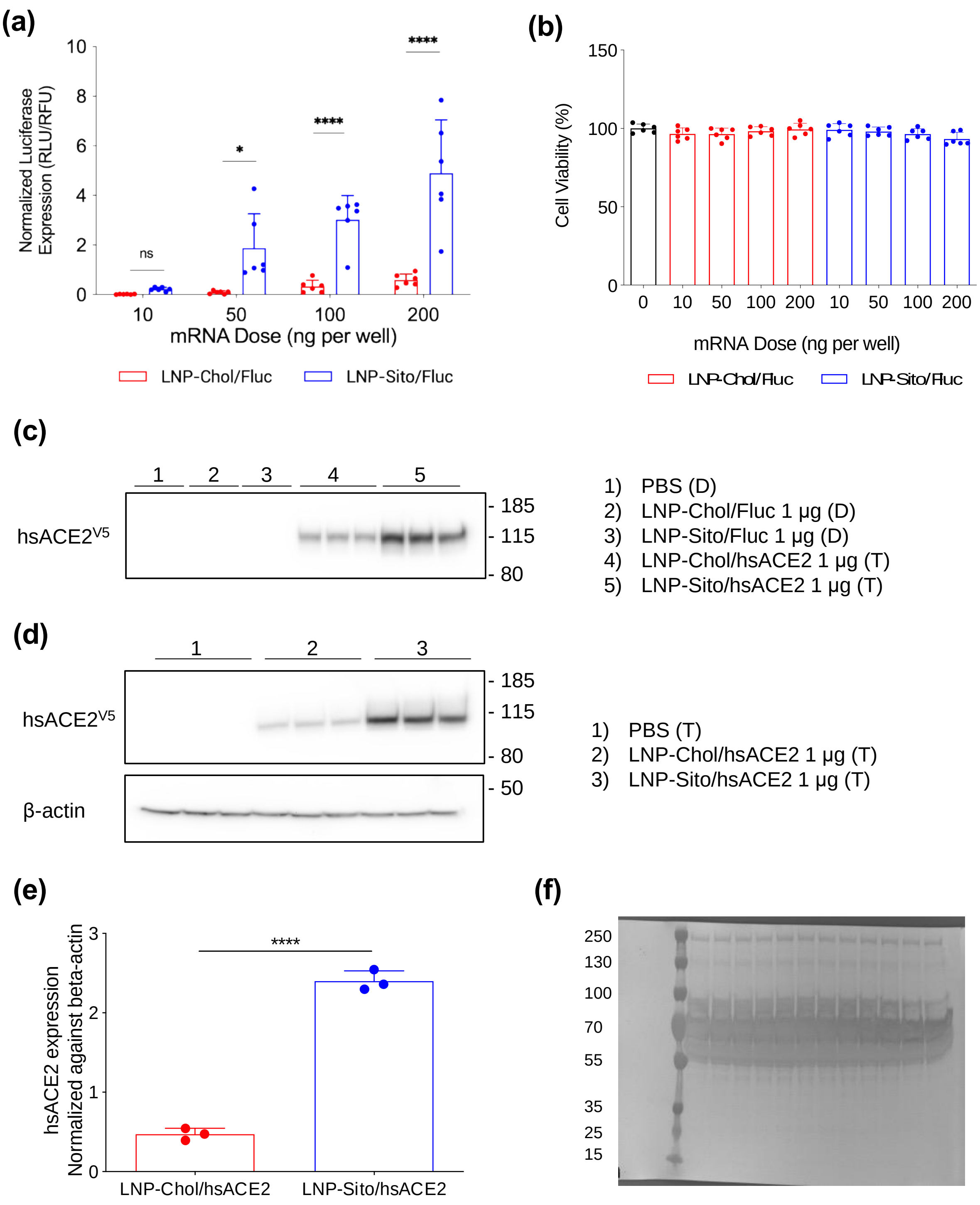


(**a**) *In vitro* luciferase and (**b**) cell viability assay of 293T cells transfected with LNP-Chol/Fluc or LNP-Sito/Fluc for 24 h (10-200 ng mRNA per well, n=6). Statistical analysis was performed using Two-way ANOVA with Sidak's multiple comparison tests. ****$p$<0.0001; *$p$<0.05; ns (not significant). (**c**) Western blot with cell-free media and (**d**)

cell lysates of 293T cells after mRNA transfection using various LNPs. Treatment and mRNA dose are described on the right of each blot. (**e**) Expression of hsACE2 protein in the 293T cell lysates using LNP-Chol/hsACE2 (red) or LNP-Sito/hsACE2 (blue) was normalized to the expression of β-actin by densitometry. (**f**) The blot used to probe the Figure 2a was stained with Coomassie blue to visualize total protein levels. Statistical analysis was performed using an unpaired t-test. ****$p<0.0001$. All data were expressed as the mean ± S.D.

**Figure S4: Nanoparticle-delivered mRNA transfection in Hep G2 cells**

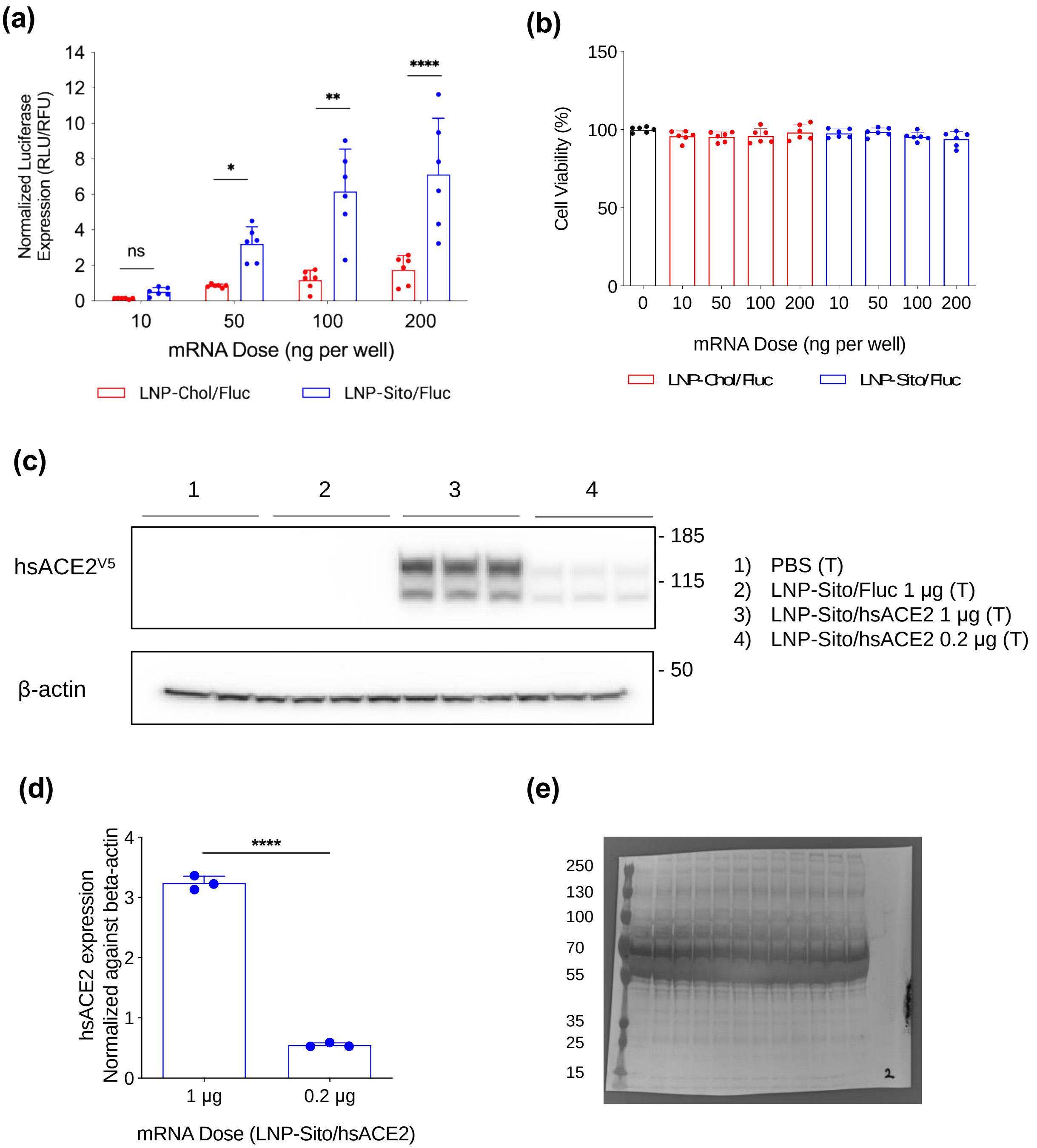


(**a**) *In vitro* luciferase and (**b**) cell viability assay of Hep G2 cells transfected with LNP-Chol/Fluc (red) or LNP-Sito/Fluc (blue) for 24 h (10-200 ng mRNA per well, n=6). Statistical analysis was performed using Two-way ANOVA with Sidak's multiple comparison tests. ****$p$<0.0001; *$p$<0.05; ns (not significant). (**c**) Western blot with cell lysates of Hep G2 cells after mRNA transfection using various LNPs. Treatment and mRNA dose are described on the right of each blot (T; n=3). (**d**) Expression of hsACE2

protein in the Hep G2 cell lysates was normalized to the expression of β-actin by densitometry. All data were expressed as the mean ± S.D. (**e**) The blot used to probe the Figure 2b was stained with Coomassie blue to visualize total protein levels. Statistical analysis was performed using an unpaired t-test. ****$p$<0.0001. All data were expressed as the mean ± S.D.

**Figure S5: *In vivo* expression of circulating hsACE2 protein post- intravenous administration of LNP-Sito/hsACE2**

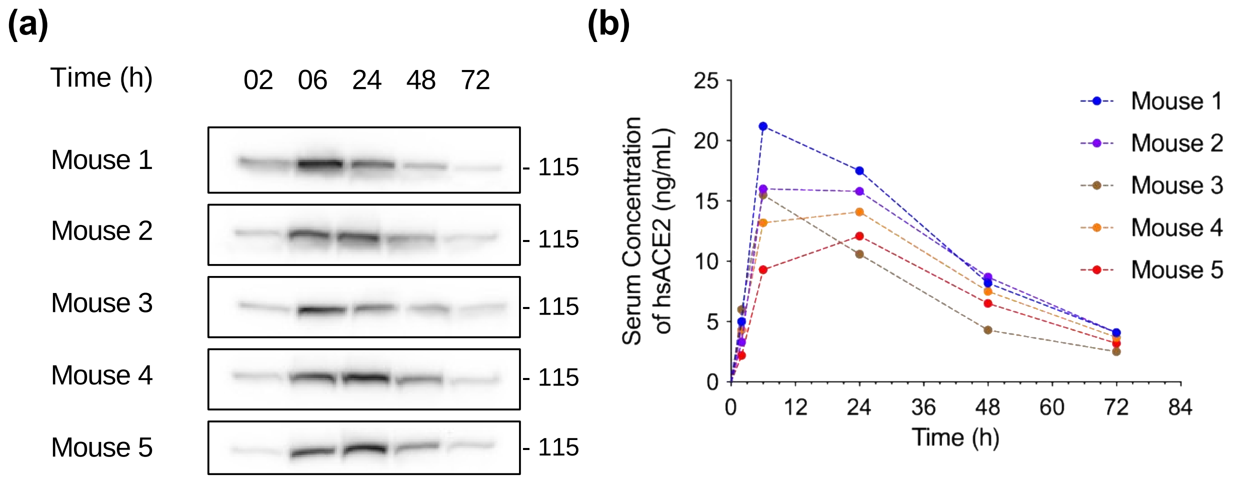


**(a)** Western blot with mouse sera collected with predetermined time intervals after IV injection of LNP-Sito/hsACE2 (n=5). **(b)** Expression kinetics of the circulatory hsACE2 protein in each mouse.

**Figure S6: LNP-delivered mRNA transfection in Calu-3 cells**

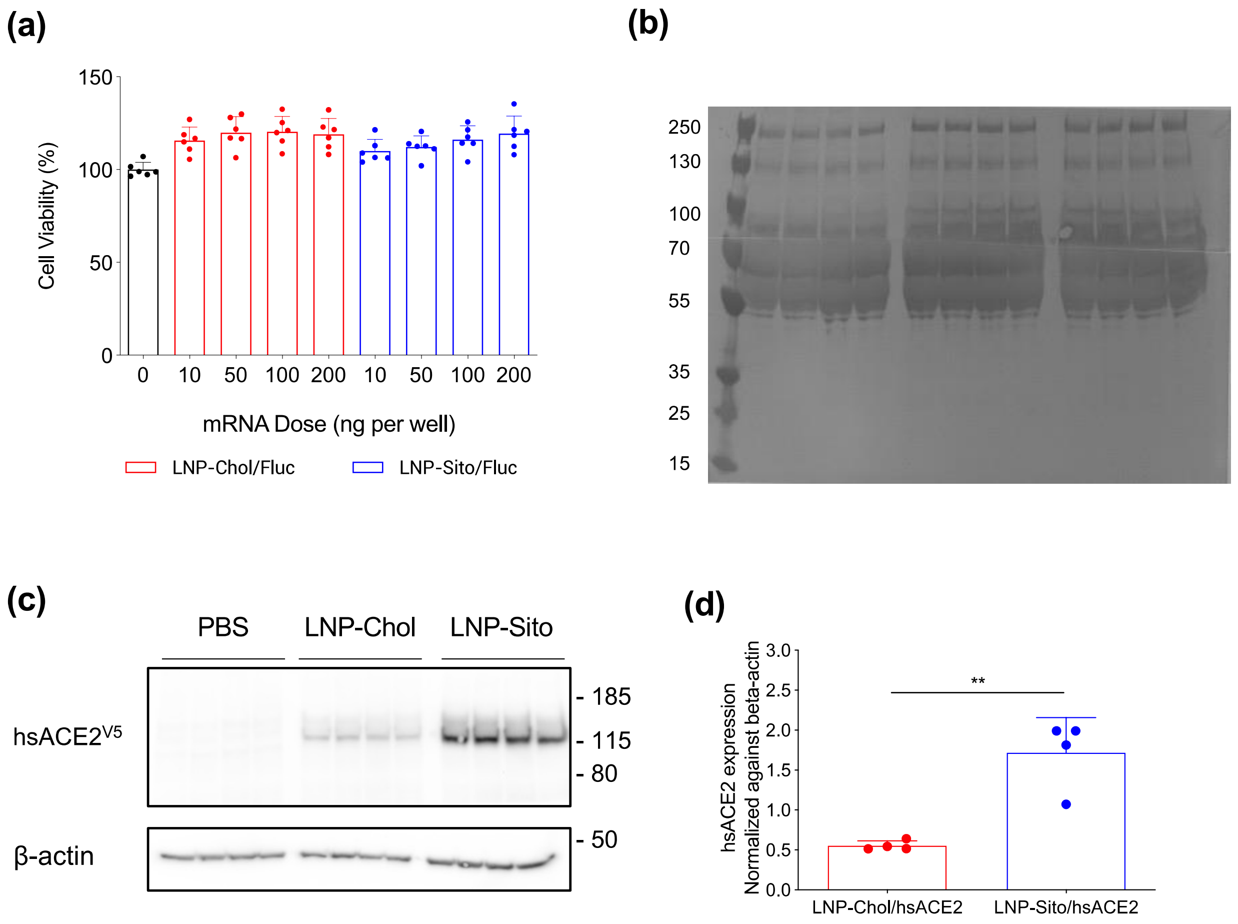


(**a**) Cell viability of Calu-3 cells transfected with LNP-Chol/Fluc (red) or LNP-Sito/Fluc (blue) for 48 h (10-200 ng mRNA per well, n=6). (**b**) The blots used to probe the Figure 3b were stained with Coomassie blue to visualize total protein. (**c**) Western blot with cell lysates of Calu-3 cells after mRNA transfection using LNP-Chol/hsACE2 and LNP-Sito/hsACE2 (n=4). Lysates of Calu-3 cells treated with PBS were used as an internal control. (**d**) Expression of hsACE2 protein in Calu-3 cell lysates using LNP-Chol/hsACE2 (red) or LNP-Sito/hsACE2 (blue) was normalized to the expression of β-actin by densitometry. Statistical analysis was performed using an unpaired t-test. ***p*<0.01. All data were expressed as the mean ± S.D.

**Figure S7: Nebulization of LNPs to deliver mRNA to mice via inhalation.**

(a)

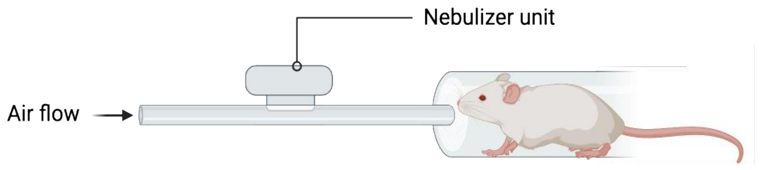


(b)

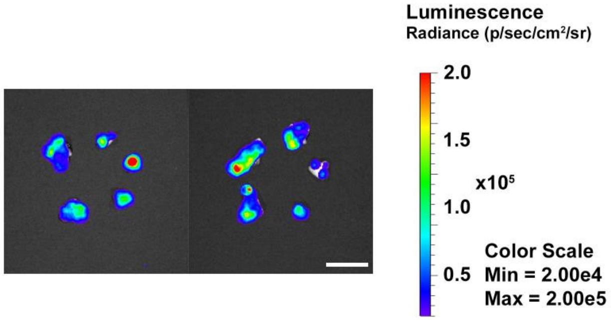


(**a**) A schematic of *in vivo* inhalation system for LNP nebulization. (**b**) *Ex vivo* bioluminescence in mouse lung lobes imaged after 24 h post-inhalation of LNP-Sito/Nluc at a dose of 100 µg mRNA per mouse. A scale bar represents 1 cm.

**Figure S8: Hematoxylin and eosin (H&E) staining of the mouse liver tissues after intravenous administration of PBS or LNP-Sito/hsACE2.**

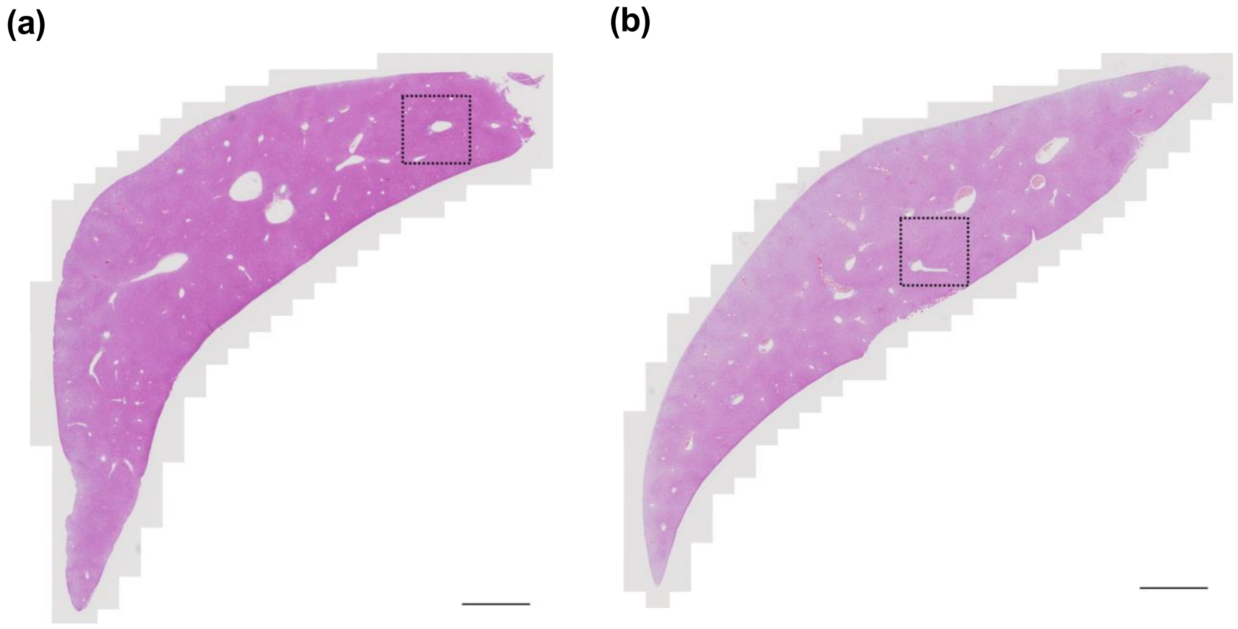


(**a,b**) H&E stained mouse liver tissues of (**a**) PBS and (**b**) LNP-Sito/hsACE2 treated groups. Black boxes represent the areas used for the Figure 4c,d. Scale bars show 1 mm. Magnification; 20X.

**Figure S9: H&E staining of the mouse lung tissues after inhalation of PBS or LNP-Sito/Nluc.**

(a)

(b)

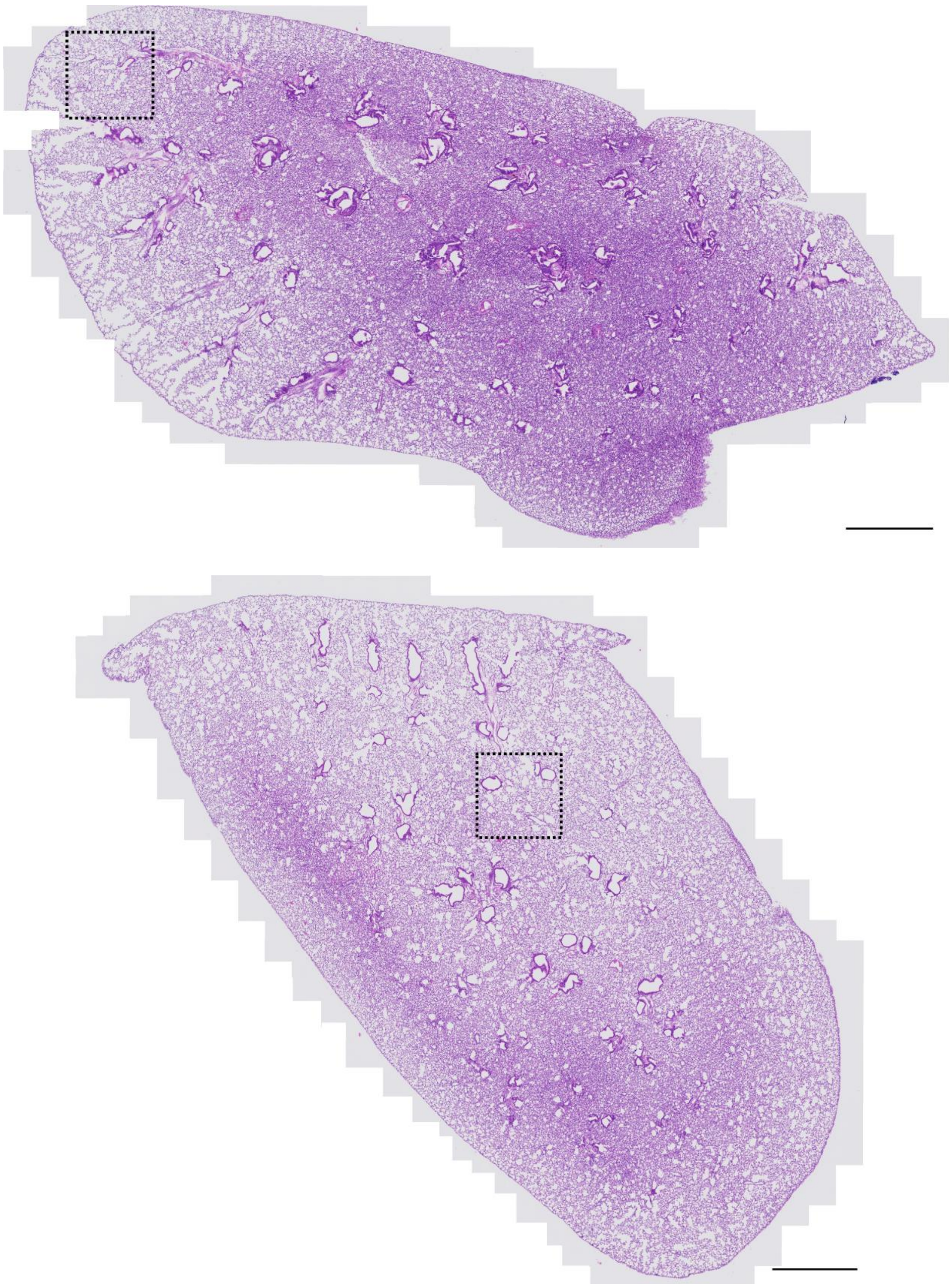

(**a,b**) H&E stained mouse lung tissues of (**a**) PBS and (**b**) LNP-Sito/Nluc treated groups. Black boxes represent the areas used for the Figure 4e,f. Scale bars show 1 mm. Magnification; 20X.

**Figure S10: Gating strategy for the detection of macrophages and neutrophils in BALF.**

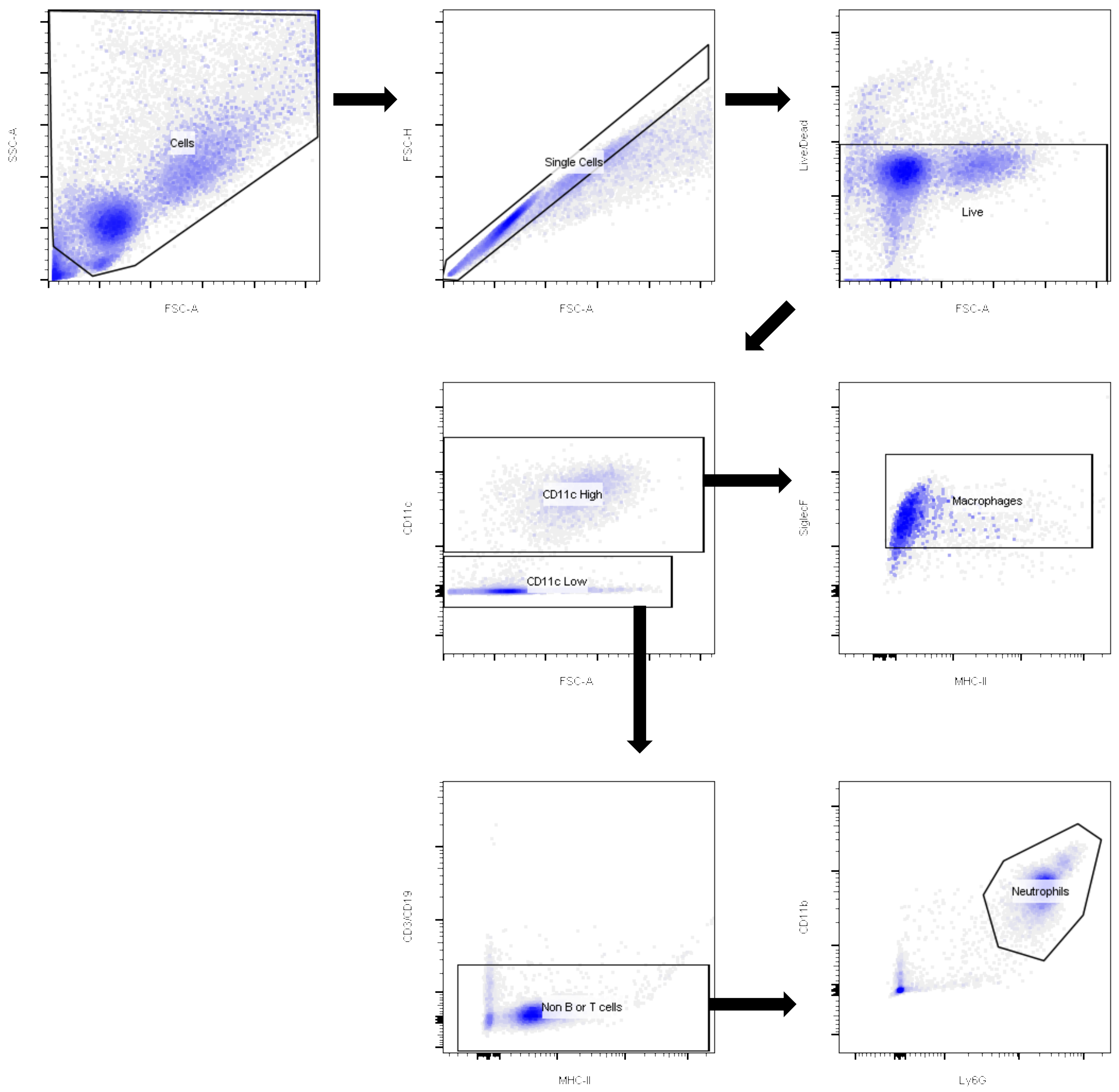


Gating was based on the BAL cells collected from LPS-challenged mouse lungs. Macrophages were gated based on SiglecF expression from CD11c$^{high}$ populations. Neutrophils were gated based on CD11b and Ly6G expression from non-B or -T cell populations.

**Figure S11: Generation of 293T-hACE2 cells and effects of hACE2 in transduction of the spike pseudovirus.**

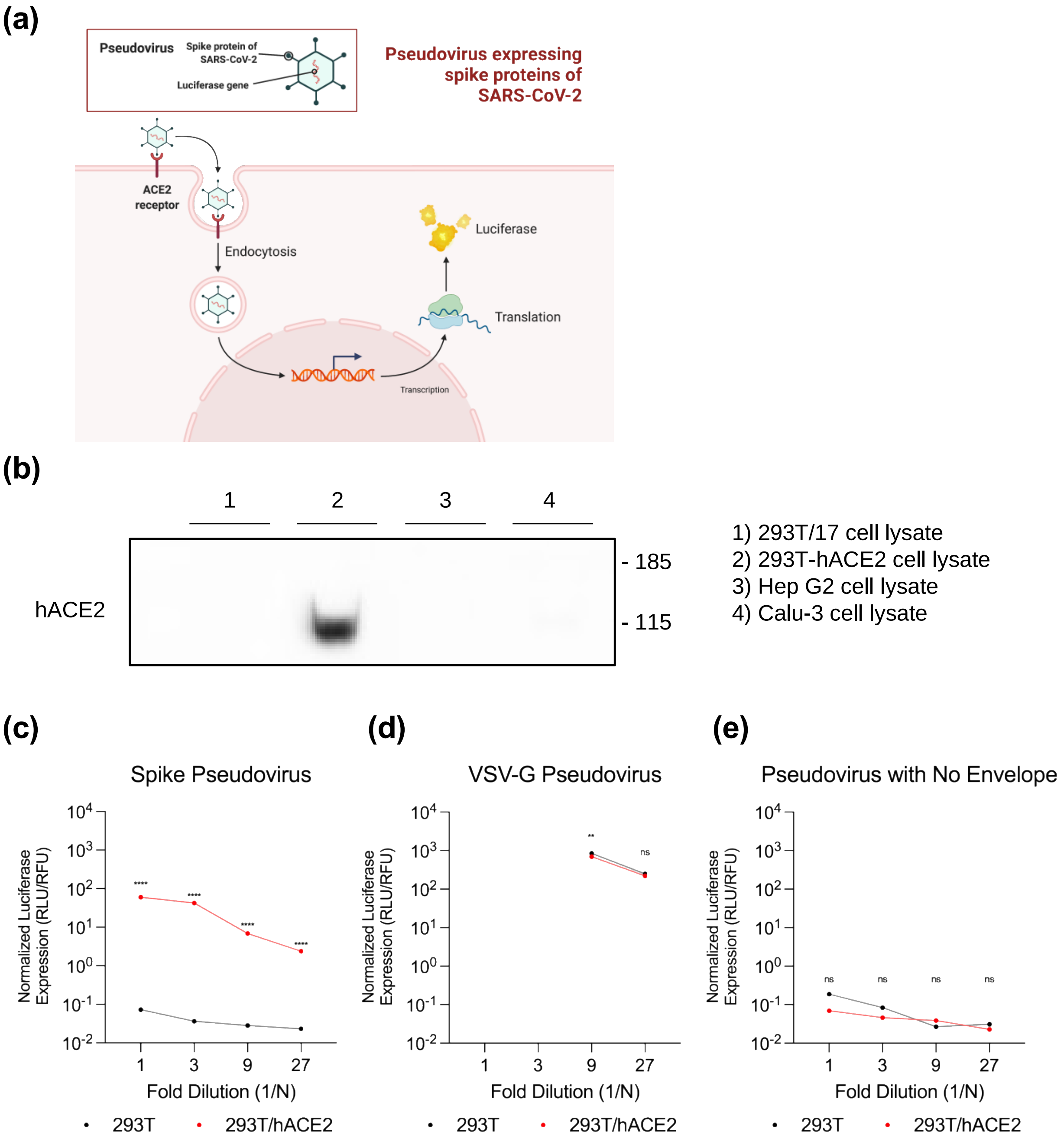


(**a**) A schematic of *Fluc*-packaged pseudovirus neutralization assay. (**b**) Western blot of hACE2 in various cell lysates. (**c-e**) Titration of (**c**) spike pseudovirus, (**d**) pseudovirus with VSV envelope glycoprotein (VSV-G), and (**e**) pseudovirus without envelope in 293T cells (black) and 293T-hACE2 (red) cells (n=3). Normalized luciferase expressions of VSV-G pseudovirus at 1 and 3-fold-dilutions were not presented due to saturation of signal. All data were expressed as the mean ± S.D. Statistical analysis was performed

using Two-way ANOVA with Sidak's multiple comparison tests. ****$p<0.0001$; **$p<0.01$; ns (not significant).

**Figure S12: Effects of hsACE2 in VSV-G pseudovirus transduction**

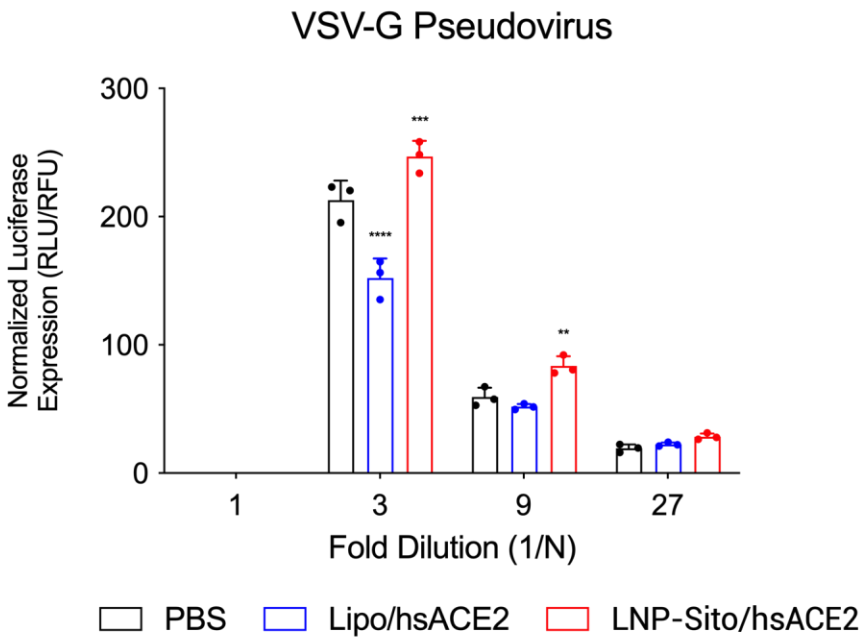


Pseudovirus displaying the VSV envelope glycoprotein (VSV-G) were incubated with various conditioned media in 293T-hACE2 cells: PBS (black), hsACE2 mRNA-loaded lipofectamine 3000 (blue), and LNP-Sito/hsACE2 (red). Pseudoviruse was serially diluted for treatment, and normalized luciferase expression was measured (n=3). Normalized luciferase expressions of VSV-G pseudovirus at 1-fold dilution were not presented due to saturation of signal. All data were expressed as the mean ± S.D. Statistical analysis was performed using Two-way ANOVA with Sidak's multiple comparison tests. ****$p$<0.0001; ***$p$<0.001; **$p$<0.01 compared to PBS-treated group.

**Table S1: Blood Chemistry Findings of the harvested sera.**

| Parameter | PBS | LNP-Sito/Fluc | LNP-Sito/hsACE2 |
|---|---|---|---|
| ALP (U/L) | 86.6 ± 7.9 | 95.2 ± 7.0 | 91.8 ± 6.6 |
| AST (U/L) | 99.0 ± 38.0 | 112.4 ± 68.9 | 167.6 ± 83.9 |
| ALT (U/L) | 19.8 ± 4.3 | 20.4 ± 3.3 | 30.0 ± 7.1 |
| BUN (mg/dL) | 20.4 ± 2.1 | 16.4 ± 2.1 | 19.8 ± 1.9 |
| GGT (U/L) | 0 ± 0 | 0 ± 0 | 0 ± 0 |
| Albumin (g/dL) | 2.80 ± 0.07 | 2.92 ± 0.08 | 2.88 ± 0.13 |
| Total Bilirubin (mg/dL) | 0.16 ± 0.05 | 0.14 ± 0.05 | 0.18 ± 0.04 |
| Total Protein (g/dL) | 4.58 ± 0.13 | 4.78 ± 0.23 | 4.74 ± 0.23 |
| Globulin (g/dL) | 1.78 ± 0.08 | 1.86 ± 0.17 | 1.86 ± 0.11 |
| Creatinine (mg/dL) | 0.1 ± 0.0 | 0.1 ± 0.0 | 0.1 ± 0.0 |

Data were presented as mean ± standard deviation (n=5).

**Table S2: Histopathological Findings of the harvested livers.**

| | **PBS-treated** | | | **LNP-treated** | | |
|---|---|---|---|---|---|---|
| **Parameter** | # Abnormal | Mean Group Score | Mean Lesion Score | # Abnormal | Mean Group Score | Mean Lesion Score |
| Infiltrate, lymphocytic, portal | 2 | 0.7 | 1.0 | 1 | 0.3 | 1.0 |
| Artifact | 0 | 0 | | 0 | 0.0 | |
| Sum-Scores: | 2 | 0.7 | 1.0 | 1 | 0.3 | 1.0 |
| No. Significant Findings | 1 | | | 2 | | |

The Mean Group Score for a tissue change is the mean of severity scores for all animals in the group, including animals for which a tissue change was not observed. The Mean Lesion Score is the mean of severity scores only for animals with a scored tissue change in the Group. Mean Lesion Scores do not include animals for which a tissue change was not observed. Data were obtained from three liver samples per group (n=3).

**Table S3: LDH activity in the collected BALF**

| Groups | LDH (U/L) |
|---|---|
| PBS-treated | 0±0 |
| LNP-treated | 0±0 |

**Table S4: List of antibodies and reagents used for flow cytometry**

| Name | Manufacturer | Catalog number |
|---|---|---|
| CD16/CD32 | Invitrogen | 14-0161-82 |
| CD11b, eFluor™ 450 | Invitrogen | 48-0112-80 |
| CD3, FITC | Invitrogen | 11-0032-82 |
| CD19, FITC | Invitrogen | 11-0193-81 |
| MHC-II, PE | Invitrogen | 12-5321-82 |
| Siglec-F, PE-CF594 | BD Biosciences | 562757 |
| Ly6G | Invitrogen | 25-9668-82 |
| CD11c, APC | Biolegend | 117310 |
| Fixable Viability Dye, eFluor™ 780 | Invitrogen | 65-0865-14 |
| UltraComp eBeads™ Compensation Beads | Invitrogen | 01-2222-41 |
